# Major adverse cardiovascular events, morbidity, and mortality, among people living with and without HIV in two northern Uganda hospitals

**DOI:** 10.1186/s12879-026-12895-6

**Published:** 2026-02-14

**Authors:** Mark Okwir, Emmy Okello, Abigail Link, Immaculate Akullo, Pancras Odongo, Bernard Omech, Francis Kiweewa, Yu Liu, David Meya, Paul R. Bohjanen, Robert C. Block

**Affiliations:** 1 Department of Public Health Sciences, University of Rochester Medical Centre, 265 Crittenden Boulevard CU420644, Rochester, NY 14642 USA; 2 Department of Internal Medicine, Faculty of Medicine, Lira University, Lira, Uganda; 3 Department of Medicine, School of Medicine, Makerere University, Kampala, Uganda; 4 Department of Medicine, Lira Regional Referral Hospital, Lira, Uganda; 5 Infectious Diseases Institute, Makerere University, Kampala, Uganda; 6 Uganda Heart Institute, Mulago, Kampala Uganda; 7 MARA Rural Health Consortium, Lira City, Uganda; 8 Department of Medicine, Division of Infectious Diseases and International Medicine, University of Rochester, New York, USA

**Keywords:** Major adverse cardiovascular events, Heart failure, Mortality, Myocardial infarction, Stroke, HIV, Prevention, Uganda

## Abstract

**Background:**

Cardiovascular disease (CVD) morbidity and mortality are increasing globally, including among patients living with human immunodeficiency virus (HIV). We aimed to determine the risk of all-cause mortality and morbidity in patients with CVD, comparing those with and without HIV infection, and whether mortality differed by HIV status during hospitalization for Major Adverse Cardiovascular Events (MACE) components (stroke, acute myocardial infarction (AMI), and heart failure) in northern Uganda.

**Methods:**

We conducted a retrospective cohort study at two hospitals in northern Uganda, comparing outcomes in CVD patients with and without HIV hospitalized from January 2015 to June 2023. We utilized a logistic regression model for crude, adjusted, and stratified analyses of MACE components and mortality by HIV status.

**Results:**

Among 2,127 CVD patient records analyzed, 292 (13.7%) had HIV. During hospitalization, 26.0% of patients living with HIV (PLWH) died compared with 15.8% of those without HIV. Similarly, among patients who experienced MACE, mortality was higher in PLWH than in those without HIV, including heart failure (38.1% vs. 22.9%), AMI (60.0% vs. 36.5%), and strokes (41.8% vs. 27.8%). In multivariable analyses, patients with cardiovascular disease living with HIV had higher odds of all-cause mortality compared with patients without HIV (aOR: 2.30, 95% CI: [1.32, 3.98], *p =* 0.003). Among individual MACE components, the odds of mortality were higher among those living with HIV, including heart failure (aOR: 3.06, CI: [1.56, 6.32], *p =* 0.001), AMI (aOR: 9.07, CI: [4.55, 18.77], *p* < 0.001), and stroke, (aOR: 2.5, CI: [1.15, 5.42], *p =* 0.02). We assessed whether HIV modifies the relationship between MACE and odds of mortality, interaction terms (example, HIV*Stroke) showed no statistical evidence of effect modification, with HIV-interaction term *p*-values: heart failure (*p* = 0.465), stroke (*p* = 0.613), and AMI (*p* = 0.615).

**Conclusion:**

All-cause mortality risk was higher among CVD patients living with HIV compared to those without. Although patients living with HIV who experienced MACE had higher in-hospital mortality, there was no statistical evidence of effect modification by HIV status. Further research is needed to understand the underlying pathophysiological mechanisms and mortality risks among patients hospitalized with MACE, including both medium- and long-term post hospitalization.

**Supplementary Information:**

The online version contains supplementary material available at 10.1186/s12879-026-12895-6.

## Introduction

Globally, approximately 40.8 million [37.0–45.6 million] people were living with the human immunodeficiency virus (HIV) at the end of 2024, with 1.3 million [1.0–1.7 million] new infections in 2024, and two-thirds being residents of sub-Saharan Africa [1, 2]. The advances in HIV management and antiretroviral therapy (ART) use have contributed to the decrease in HIV-related morbidity and mortality globally [3]. Despite this success, 490,000 to 820,000 people still died in 2024 from HIV-related causes [4]. The major cause of death in people living with HIV (PLWH) is non-communicable disease, largely from cardiovascular diseases (CVD) [4, 5]. As a result, it is estimated that by 2030, 73.0% of individuals living with HIV will be 50 years and older, and 78.0% of individuals living with HIV will have cardiovascular disease [6]. 

Cardiovascular disease is common in HIV infection [7], and the majority of individuals with the two comorbidities are hospitalized with major adverse cardiac events. The global burden of HIV-associated cardiovascular diseases has tripled over the past two decades [8]. An estimated 17.9 million CVD deaths occur annually [5], and 77.0% of these deaths occur in low- and middle-income countries [9]. This is because individuals with HIV have a higher risk of major adverse cardiovascular events and all-cause mortality [10]. These events include acute myocardial Infarction (AMI) [7, 11], stroke [12–15], heart failure [16, 17], and cardiovascular death [17–19]. Many studies define MACE differently in their reporting [16]. In this study, we used the most commonly recognized three-point definition, which includes heart failure, acute myocardial infarction, and stroke [16]. The mechanisms explaining the risks include direct HIV viral effects and related opportunistic infections [20, 21], persistent inflammation by HIV [22], direct endothelial injury [23], extracellular-vesicle-mediated vascular damage [24], premature aging [25], and atherosclerosis in individuals [26, 27]. Hypertension is a major risk factor for heart failure, AMI, and stroke, and it is more common among people living with HIV than those without HIV [28–30]. Traditional cardiovascular disease risk factors like smoking, combined with HIV infection and prolonged antiretroviral therapy further exacerbate this risk [21, 31, 32]. Despite the expanded availability of ART and statins in resource-limited settings, disparities in cardiovascular disease outcomes persist when compared with high-income countries [33]. These major adverse cardiovascular event outcomes following hospitalization remain poorly studied in sub-Saharan Africa [34]. 

Furthermore, it is uncertain whether mortality risk differs between individuals with and without HIV who are hospitalized with major adverse cardiac events. However, some studies show that individuals living with HIV face up to a 50.0% greater risk of adverse events compared to those without HIV [35–38]. This increased morbidity risk persists even among those on ART [39]. It is unclear if the presence of HIV infection in individuals hospitalized with major adverse cardiovascular events (stroke, AMI and heart failure) could modify the risk of in-hospital mortality. This uncertainty raises questions about whether prevention should focus on pre-hospitalization strategies, on improving care for hospitalized patients, or whether MACE-related mortality risk differs by HIV status. Further research is needed to understand these morbidity and mortality differences. However, unique prevention strategies require studies from these subgroups, which are largely lacking. In the sub-Saharan region, there are limited prospective studies on major adverse cardiac events, despite the high morbidity and mortality associated with HIV exposure and cardiovascular disease. For example, high-income countries contributed to 81.1% of all the global cardiovascular research, and contributed to 8.5% of global death rates, while low-income countries with 57.0% of deaths, contributed to only 2.8% of cardiovascular research [40]. This emphasizes the need for further studies in these low-income regions in those with high cardiovascular disease and mortality risk.

We aimed to assess all-cause mortality and the odds of death from major adverse cardiovascular events (stroke, AMI, and heart failure) among patients with and without HIV hospitalized in two regional referral hospitals in northern Uganda. Additionally, the study assessed whether HIV infection modified the association between major adverse cardiovascular events and all-cause mortality during hospitalization. Thus, the study hypothesized that among patients hospitalized with major adverse cardiovascular events, comorbidity with HIV infection increases and modifies the risk of in-hospital mortality. Here we report on the outcomes of this retrospective cohort study which utilized data from two hospitals.

We provide evidence for: (a) the association between CVD patients living with and without HIV and odds of all-cause mortality; (b) the association between individual components of MACE (heart failure, AMI, and stroke) and odds of all-cause mortality, comparing patients living with and without HIV; and (c) effect modification by HIV status in the relationship between MACE and all-cause mortality. Effect modification is to assess whether the combined (joint) effect of HIV and MACE on mortality differs from what would be expected from their individual effects, indicating evidence of biological synergistic or antagonistic interaction on either the additive or multiplicative scale [41]. We discuss these results and their implications for the prevention of CVD mortality in the context of HIV infection, cardiovascular risk, and major adverse cardiac events.

## Methods

### Study population and setting

This retrospective cohort study assessed the risk of all-cause mortality and associated factors. It also examined the association between individual components of major adverse cardiovascular events (MACEs) and mortality among 2,127 patients hospitalized with cardiovascular disease, with or without HIV infection. We further evaluated if mortality among patients with MACE was different by HIV status (effect modification). Patient records were accessed from July 12, 2022, to June 12, 2023, and again from July 21, 2023, to July 21, 2024, following an extension. The study population was composed of all patients hospitalized for any cardiovascular disease diagnoses at Lira Regional Referral Hospital (LRRH) and Lira University Hospital (LUH) between January 2015 to June 2023. We included all male and female adult patients aged 18 years and above, with confirmed HIV status, and any clinical diagnosis of CVD at the time of hospitalization. Patients were excluded if they had missing or ineligible records, died on arrival, had no documentation on CVD diagnosis, HIV status, or hospital outcomes/deposition status. Hospitalized patients had similar referrals, diagnoses, treatments, and records in these government-owned facilities. They received free treatment, ensuring unhindered access to healthcare and minimizing the likelihood of potential selection bias.

In northern Uganda, LRRH and LUH tackle the region’s high HIV and CVD burden. LRRH, a 350-bed government-owned hospital located 340 km from Kampala, had 232,014 outpatient visits and 12,203 inpatient admissions in 2022 [42]. LUH, a 110-bed teaching hospital 11 km from Lira City center, focuses on special medical outpatients. Together, they serve a population of 2.3 million Ugandans as referral hubs for the district and beyond. Lira hospital has the region’s largest HIV chronic care clinic and manages over 14,000 active patients living with HIV on ART. It has a larger cardiac clinic with a team of clinicians previously trained in echocardiography [43]. These two institutions have medical record departments, which we utilized as our data source. The data were captured without prior knowledge of the study exposure and outcome by the study team. We extracted demographic and clinical data using a structured data entry questionnaire by intern doctors, assisted by records officers who identified the files.

### Major adverse cardiovascular events exposure and HIV status

The primary exposures are components of major adverse cardiovascular events (Stroke, Heart Failure, and AMI), all of which were coded as binary variables. We assessed HIV status among CVD patients by reviewing HIV testing and diagnosis records, among ART– naïve and ART– experienced patients. For HIV tests, we used national and World Health Organization testing and treatment guidelines [44, 45]. All patients’ files routinely included a special identification for HIV tests and results. However, patients already in care before hospitalization had ART clinic-identification numbers on special cards. The Uganda national guidelines require routine HIV testing for all hospitalized patients, so HIV status records were readily available. Cardiovascular disease assessment involves the use of clinical, laboratory, cardiac scans, and radiological records. Some of these tests were not routinely performed in these hospitals. However, as part of a study and the Uganda Heart Institute program, “Improving the Accuracy of Heart Failure Diagnosis in Low–Resource Settings Through Task Sharing and Decentralization”, relatively accurate cardiac diagnostic tests were available [43]. We also included prior cardiac assessments (documented on the referral form) in the evaluation. AMI and congestive heart failure were diagnosed based on clinical assessment, positive cardiac biomarkers, electrocardiogram (ECG), and transthoracic echocardiography. AMI was defined by chest pain radiating to the arm, shortness of breath, sweating, fatigue, abnormal ECG (Q-waves, ST-segment changes), elevated cardiac enzymes (troponin, creatine kinase if available), or echocardiographic wall motion abnormalities. Cardiac computed tomography (CT) or magnetic resonance imaging (MRI) was unavailable. Heart failure was diagnosed based on clinical assessment (history, examination, blood tests, New York Heart Association staging, and radiological exams). Stroke diagnosis was also based on the clinician’s assessment, and brain-computer tomography scans (CT scans) when available. Stroke was defined clinically as a rapid onset of clinical signs of focal or global cerebral dysfunction, symptoms lasting more than 24 h or leading to death, and no apparent cause of vascular origin. Stroke was classified as ischemic or hemorrhagic.

### All-cause mortality outcomes

The primary outcome in this study was all-cause hospital mortality in patients with stroke, AMI, and heart failure, hospitalized with CVD with or without HIV exposure. All-cause mortality, coded as a binary variable was ascertained by reviewing clinical records, discharge registers, hospital death registries, and certificates. We extracted dates of admission and death, medical diagnoses, and cause of death from patients’ files, discharge notes, death certificates, and reports from the pathology department. Survival status after patients’ discharge was not ascertained because we were unable to trace them to post–hospitalization, and it was beyond the scope of the study period. None of the data assistants involved in extracting data were aware of the study’s exposure or outcomes.

### Covariates

We included lifestyle risks for CVD, including cigarette smoking (categorized as former, current, or non-smokers), alcohol consumption defined as a binary variable, family history of CVD, prior cardiac diseases, kidney disease, diabetes mellitus, dyslipidemia, laboratory results, diagnoses, and reasons for previous hospitalization. We excluded variables, including electrolytes and medications, that were largely missing from the records. Variables used in the study were identified from prior literature. A directed acyclic graph (DAG) was used to identify the variables that would be required in the study for each component of major adverse cardiovascular events. A standardized computer-based questionnaire was used by trained data assistants (junior medical doctors) capable of reading medical details in the patients’ files. Data extraction took place within the registry building, where patient files were stored to ensure confidentiality and privacy. Other covariates included CVD diagnoses like dilated cardiomyopathy, hypertension, atrial fibrillation, and rheumatic heart disease. We ensured that the data were verified for completeness, accuracy, and consistency by checking all different entries for outliers, coding, formats, and any important missing variables like dates, sex, age, and HIV diagnoses.

### Statistical analyses

We employed an unconditional logistic regression model to examine the association between all-cause mortality in CVD patients (outcome) and each component of major adverse cardiac events (exposure). Mortality, a binary variable coded as Death = 1, and Alive = 0 (reference group). The independent variables were Stroke (Yes = 1, No = 0), Heart failure (Yes = 1, No = 0), Acute myocardial infarction (Yes = 1, No = 0), and Any MACE (Yes = 1, No = 0). The absence of these conditions was considered as the reference group. After data cleaning, and checking for coding and data completeness, we examined data for outliers, missing values, and parametric assumptions (e.g. normality and independence of observations assumptions). We performed descriptive univariate statistical analyses using the Mann-Whitney U-test, a non-parametric statistical test utilized to compare the distributions of two independent groups. We described the study population using proportions, percentages, and median (interquartile range: IQR). The incidence of all-cause mortality among CVD patients was described using chi-squared analyses. We compared the risk of mortality among patients living with HIV and those without, describing mortality outcomes, stratified by HIV status.

The estimate of association was the odds ratio (OR) and 95% confidence intervals (CI) for all-cause mortality, because the prevalence of death (binary outcome) was assumed to be less than 10%. We regressed mortality against each component of the MACE (primary exposure) and several other covariates among CVD patients. Covariates were classified as binary or categorical (e.g., smoking categories). We adjusted for confounders in multivariable regressions. These confounders were selected a priori based on existing literature, and a directed acyclic graph (DAG) presented in supplementary Figures S1–S3. In addition, we used a 10% change in effect estimates (OR) to further identify confounders. We regressed mortality with each component of MACE and several covariates as linear predictors. Crude and adjusted OR were estimated for each component of the MACE (exposure) models. Each model was fitted with their minimum sufficient set of confounders. Confounders modeled included age, sex, smoking, diabetes, alcohol consumption, chronic kidney disease, dyslipidemia, hypertension, and history of cardiac disease. In all the analyses, statistical significance was defined as *a p*-value of < 0.05 and a 95% confidence interval (5% two-sided alpha).

Next, we performed stratified analyses for each model for components of MACE (subgroups) to evaluate whether the effect of each composite component of MACE (stroke, AMI, and heart failure) on all-cause mortality differs by HIV status (HIV-positive/ negative) among CVD patients hospitalized with these adverse events. HIV interaction terms were included in the models (e.g. Stroke*HIV) to evaluate effect modification. For each model, a significant *p*-value was interpreted as mortality being different between patients with and without HIV and MACE. We further reported the stratified results by HIV status to demonstrate effect modification. Patients with missing data (48/2,175; 2.2%) were excluded during data collection and analyses, this was because data were missing at random. We performed all data management and analyses using SAS (version 9.4; SAS Institute Inc., Cary, NC) and R (version 4.1.3 R foundation for statistical computing, Vienna, Austria).

## Results

### Study population characteristics

Characteristics of the study population, grouped by HIV status, are shown in Table 1. Of the 2,127 hospitalized patients with any cardiac illness within the study period, 292 patients (13.7%) were confirmed with HIV, while 1,835 patients were HIV seronegative. Most patients were female (60.5%), reflecting healthcare utilization and hospitalization patterns, with a median age of 63 years [Interquartile range, IQR:45–75 years]. CVD patients living with HIV were generally younger (median age = 51 years [IQR: 37–67 years] compared to CVD patients without HIV (median = 65 years [IQR: 47–77 years]). In total, 52.4% of the patients had a history of cardiac disease, and 728 (34.2%) patients used antiplatelet drugs. Rheumatic heart disease was present in 192 patients (12.8%), of whom 170 (88.5%) were HIV–negative. Overall, 23.1% of patients with CVD were former or current smokers, and only 7.1% consumed alcohol. Additionally, 15.4% of patients with CVD had diabetes mellitus (either newly diagnosed or previously known). Similarly, 16.0% of patients with CVD had a history of chronic kidney disease (CKD), with the majority (87.4%) being HIV–seronegative. Hypertension was the most common CVD morbidity at hospitalization, affecting 40.9% of patients. It was more prevalent among patients with HIV than those without (49.3% vs. 39.6%). Dyslipidemia affected 28.2% of all patients, with only 7.8% of them being patients living with HIV. Overall, 1128 of 2127 patients (53.0%) had heart failure, making it the most prevalent adverse event at hospitalization. AMI was the second most common MACE, affecting 472 patients (22.2%). It was more common among patients living with HIV (27.4%) compared to those without HIV (21.4%). Stroke was the least common MACE, with equal proportions between patients living with HIV (55 of 292;18.8%) and without (345 of 1835;18.8%).

**Table 1:** Demographic and clinical characteristics of the study population

Characteristic		ALL Patients	Patients with HIV & CVD	HIV seronegative with CVD
	Category	*N* = 2127 *n* (%)	*N* = 292 *n* (%)	*N* = 1835 *n* (%)
Demographic				
Sex	Female	1287 (60.5)	164 (56.2)	1123 (61.2)
	Male	840 (39.5)	128 (43.8)	712 (38.8)
Age median (IQR), years		63 (45–75)	51 (37–67)	65 (47–77)
< 63 years	Yes	1035 (48.7)	194 (66.4)	841 (45.8)
≥ 63 years	Yes	1087 (51.1)	98 (33.6)	989 (53.9)
Cigarette smoking	All	2127	292	1835
Former	Yes	440 (20.7)	88 (30.1)	352 (19.2)
Current	Yes	52 (2.4)	13 (4.5)	39 (2.1)
Non-Smoker	Yes	1635 (76.9)	191 (65.4)	1444 (78.7)
Alcohol consumption	Yes	150 (7.1)	33 (11.3)	117 (6.4)
CVD history	Yes	1114 (52.4)	118 (40.4)	996 (54.3)
Clinical Characteristics				
Diabetes Mellitus	Yes	328 (15.4)	35 (12.0)	293 (16.0)
Chronic Kidney disease	Yes	341 (16.0)	43 (14.7)	298 (16.2)
Dyslipidemia	Yes	600 (28.2)	47 (16.1)	553 (30.1)
Antiplatelets	Yes	728 (34.2)	56 (19.2)	672 (36.6)
Atrial fibrillation	Yes	234 (11.0)	33 (11.3)	201 (11.0)
Hypertension	Yes	871 (40.9)	144 (49.3)	727 (39.6)
Rheumatic Heart Disease	Yes	192 (9.0)	22 (7.5)	170 (9.3)
Dilated Cardiomyopathy	Yes	233 (11.0)	37 (12.7)	196 (10.7)
Heart Failure	Yes	1128 (53.0)	160 (54.8)	971 (52.9)
Acute myocardial infarction	Yes	472 (22.2)	80 (27.4)	392 (21.4)
Stroke	Yes	400 (18.8)	55 (18.8)	345 (18.8)

IQR – Interquartile Range, CVD – cardiovascular disease

### Crude mortality patterns among CVD patients with and without HIV

Patients living with HIV and diagnosed with CVD experienced a high incidence of mortality, especially following hospitalization with major adverse events. All-cause mortality incidence proportions among patients with CVD, grouped by HIV status, are shown in Table 2. We compared the distribution of mortality between patients living with and without HIV across various variables using bivariate analyses with the Chi-Squared test. The average length of hospital stay was 7 days (IQR:3–13 days). Overall, there were 366 deaths among the 2,127 patients with CVD, resulting in a mortality risk of 17.2%. Additionally, patients living with HIV and CVD had a higher mortality risk (26.0%) compared to HIV seronegative patients (15.8%). More so, the high mortality among CVD patients living with HIV was observed in those with a history of smoking, alcohol consumption, diabetes, chronic kidney disease, atrial fibrillation, hypertension, rheumatic heart disease, and dilated cardiomyopathy. There was a consistent pattern of higher mortality incidence among patients with both CVD and HIV infection compared to those without HIV. This was observed among females (25.0% vs. 14.2%) and males (27.3% vs. 18.4%). Among patients with major adverse cardiac events, this pattern persisted, including those with heart failure (38.1% vs. 22.9%), acute myocardial infarction (60.0% vs. 36.5%), stroke (41.8% vs. 27.8%), and any adverse event (38.1% vs. 17.8%), as shown in Table 2.

**Table 2:** Distribution of All-Cause mortality in CVD Patients, stratified by HIV status

ALL CVD patients	CVD Patients Living with HIV *N* = 292		CVD Patients without-HIV *N* = 1835	
	Total Patients	Deaths *n* (%)	Total Patients	Deaths *n* (%)
Sex	292	76 (26.0)	1835	290 (15.8)
Female	164	41 (25.0)	1123	159 (14.2)
Male	128	35 (27.3)	712	131 (18.4)
Age Category, years	292	76	1830	290
< 63	194	28 (14.4)	841	128 (15.2)
≥ 63	98	48 (49.0)	989	162 (16.4)
Smoking	292	76	1835	290
Yes	201	31 (15.4)	341	52 (15.2)
Alcohol	176	43	1114	157
Yes	33	07 (21.2)	117	23 (19.7)
Diabetes	292	76	1826	289
Yes	35	08 (22.9)	293	39 (13.3)
CKD	288	76	1793	280
Yes	43	16 (37.2)	298	63 (21.1)
Atrial fibrillation	203	56	765	135
Yes	33	11 (33.3)	201	32 (15.9)
Hypertension	292	75	1835	290
Yes	144	49 (34.0)	727	142 (19.5)
RHD	243	66	1254	197
Yes	22	06 (27.3)	170	26 (15.3)
DCMP	292	76	1835	290
Yes	37	08 (21.6)	196	20 (10.2)
Heart Failure	292	76	1835	290
Yes	160	61 (38.1)	968	222 (22.9)
Acute MI	292	76	1828	287
Yes	80	48 (60.0)	392	143 (36.5)
Stroke	291	75	1828	287
Yes	55	23 (41.8)	345	96 (27.8)
ANY MACE	292	76	1835	290
Yes	160	61 (38.1)	1486	265 (17.8)

RHD: Rheumatic Heart Disease, DCMP: Dilated Cardiomyopathy, AMI: Acute Myocardial Infarction﻿

MACE: Major Adverse Cardiovascular Events, CKD: Chronic Kidney Disease

### Association between HIV and risk of mortality

#### Demographic risk factors for in-hospital mortality among CVD Patients, stratified by HIV status

We assessed whether traditional risk factors for developing CVD were also associated with in-hospital mortality risk, beginning with demographic factors, as shown in Table 3(a). We examined the relative odds of all-cause mortality among CVD patients living with HIV compared to those without HIV. In unadjusted analyses, CVD patients living with HIV had 87.0% greater relative odds of mortality compared to HIV–seronegative patients. After adjusting for confounders, patients living with HIV had 130.0% greater relative odds of mortality compared to HIV-seronegative individuals (aOR: 2.30, 95% CI: [1.32, 3.98], *p =* 0.003).

Male patients with CVD had 34.0% greater relative odds of death compared to female patients (OR: 1.34, 95% CI: [1.07, 1.69], *p =* 0.012). However, the relative odds of mortality did not differ by HIV status; HIV positive (aOR: 1.08, 95% CI: [0.63, 1.84]) versus (aOR: 1.41, 95% CI: [1.09, 1.82]) for the HIV negative group. Age, when categorized as younger or older than the median (63 years), was not significantly associated with in-hospital mortality (aOR: 1.24, CI: [0.98, 1.58], *p =* 0.068).

Patients who smoked had 2.09 times the odds of mortality compared to non-smokers after adjusting for confounders (aOR: 2.09, 95% CI: [1.21, 3.59], *p =* 0.007). When stratified by HIV status, CVD patients living with HIV who smoked had 3.68 times the odds of death compared to non-smokers (aOR: 3.68, 95% CI: [1.42, 12.37]). Additionally, patients with a prior history of CVD had 59.0% greater relative odds of death compared to those without, but this was statistically nonsignificant (*p =* 0.062), even after stratification by HIV status.

**Table 3(a):** Crude and adjusted odds ratio (and 95% CI) of All-Cause mortality and the associated demographic factors among CVD Patients, stratified by HIV status

Models	Crude Analyses (All CVD Patients) (*N* = 2127)		Adjusted Analyses (All CVD Patients) (*N* = 2127)		Stratification	
					CVD Patients Living with HIV **(** ***N*** ** = 292)**	CVD Patients without-HIV **(** ***N*** ** = 1835)**
	**OR [95% CI]**	***p-V*** **alue**	**OR [95% CI]**	***p-*** **Value**	**OR [95% CI]**	**OR [95% CI]**
Sex ^A^						
Male	1.34 [1.07,1.68]	< 0.001	1.34 [1.07, 1.69]	0.012	1.08 [0.63, 1.84]	1.41 [1.09, 1.82]
Age, years ^B^						
≥ 63	1.03 [0.83, 1.30]	0.772	1.24 [0.98, 1.58]	0.068	1.39 [0.78, 2.48]	1.22 [0.94, 1.58]
Alcohol ^C^						
Yes	1.43 [0.91, 2.17]	0.107	0.95 [0.46, 1.88]	0.898	0.52 [0.12, 1.89]	1.33 [0.54, 3.01]
Smoking ^D^						
Smoker	0.95 [0.83, 1.10]	0.508	2.09 [1.21, 3.59]	0.007	3.68 [1.42, 12.37]	1.44 [0.66, 2.89]
HIV – Status ^E^						
Yes	1.87 [1.40, 2.50]	< 0.001	2.30 [1.32, 3.98]	0.003	-	-
CVD History ^D^						
Yes	0.92 [0.73, 1.15]	0.442	1.59 [0.98, 2.60]	0.062	2.49 [0.92, 7.08]	1.35 [0.76, 2.44]

A Adjusted for: HIV-status, Age, and Cigarettes Smoking (CS)

B Adjusted for: HIV-status, Sex, CS, Hypertension (HT), and Diabetes Mellitus (DM)

C Adjusted for: Age, Sex, HIV-status, CS, Dyslipidemia, HT, and DM

D Atrial fibrillation adjusted for: Age, Sex, HIV-status, Alcohol, dyslipidemia, HT, and DM

E Adjusted for: Age, Sex, CS, Alcohol, Dyslipidemia, HT, DM, and Cardiac Disease History (CD)

CI: Confidence Interval

#### Comorbidity factors associated with In-Hospital mortality among CVD Patients, stratified by HIV status

We further evaluated whether traditional comorbid factors were also associated with in-hospital mortality among patients with CVD, as shown in Table 3(b). Patients with CKD had 2.13 times the odds of death compared to those without CKD (aOR: 2.13, 95%CI: [1.20, 3.69], *p =* 0.008). This mortality differed between patients living with HIV and those without HIV, with odds ratios of 4.16 and 1.99, respectively. This suggests that the effect of CKD on mortality was modified by HIV infection. Similarly, patients with hypertension had 71.0% greater relative odds of mortality compared to non-hypertensive patients (aOR: 1.71, 95% CI: [1.35, 2.16], *p* < 0.001). The observed stratum-specific odds ratio for mortality was similar across HIV status, indicating no effect modification.

We observed 43.0% lower relative odds of mortality among patients who used antiplatelet drugs compared to non-users in adjusted analyses (aOR: 0.57, 95% CI: [0.43, 0.75], *p* < 0.001). However, the effect was not modified by the presence of HIV infection.

No significant mortality differences were observed for alcohol use, diabetes, dyslipidemia, atrial fibrillation, rheumatic heart disease, or dilated cardiomyopathy. Stratification by HIV status revealed no evidence of effect modification for these variables, indicating that their impact on mortality was consistent regardless of HIV status.

**Table 3(b):** Crude and adjusted odds ratio (and 95% CI) of All-Cause mortality and the associated comorbidity factors among CVD Patients, stratified by HIV status

Models	Crude Analyses (All CVD Patients) (*N* = 2127)		Adjusted Analyses (All CVD Patients) (*N* = 2127)		Stratification	
					CVD Patients Living with HIV **(** ***N*** ** = 292)**	CVD Patients without-HIV **(** ***N*** ** = 1835)**
	**OR [95% CI]**	***p-V*** **alue**	**OR [95% CI]**	***p-*** **Value**	**OR [95% CI]**	**OR [95% CI]**
Diabetes ^D^						
Yes	0.77 [0.55, 1.07]	0.131	1.26 [0.65, 2.30]	0.477	0.99 [0.23, 3.77]	1.33 [0.63, 2.63]
CKD ^D^						
Yes	1.59 [1.20, 2.10]	< 0.001	2.13 [1.20, 3.69]	0.008	4.16 [1.21, 14.76]	1.99 [0.99, 3.83]
Dyslipidemia ^F^						
Yes	1.17 [0.83, 1.64]	0.375	1.61 [0.97, 2.70]	0.068	1.94 [0.69, 5.44]	1.50 [0.84, 2.78]
Antiplatelets ^G^						
Yes	0.58 [0.45, 0.75]	< 0.001	0.57 [0.43, 0.75	< 0.001	0.68 [0.31, 1.37]	0.54 [0.40, 0.71]
Atrial fib ^D^						
Yes	0.89 [0.61, 1.29]	0.550	1.64 [0.77, 3.39]	0.189	1.12 [0.30, 3.85]	1.98 [0.72, 5.16]
Hypertension ^H^						
Yes	1.74 [1.38, 2.18]	< 0.001	1.71 [1.35, 2.16]	< 0.001	2.46 [1.42, 4.33]	1.57 [1.21, 2.04]
RHD ^G^						
Yes	0.92 [0.61, 1.38]	0.725	0.94 [0.45, 1.81]	0.853	0.32 [0.05, 1.25]	1.44 [0.63, 3.07]
DCMP^K^						
Yes	0.63 [0.41, 0.93]	0.027	1.02 [0.51, 1.92]	0.950	0.77 [0.19, 2.56]	1.11 [0.48, 2.34]

D Atrial fibrillation adjusted for: Age, Sex, HIV-status, Alcohol, dyslipidemia, HT, and DM

F Adjusted for: Age, Sex, CS, Alcohol, Dyslipidemia, HT, DM, CD, and chronic kidney disease (CKD)

G Adjusted for: Age, Sex, HIV-status, CS, and dyslipidemia

H Adjusted for: Age, Sex, HIV-status, CS, and DM

K Adjusted for: Age, Sex, HIV-status, CS, dyslipidemia, and alcohol

CI: Confidence Interval

### Association between MACE and Mortality, and effect modification by HIV

We conducted an adjusted multivariable analysis to assess the odds of mortality associated with major adverse cardiac events, stratified by HIV status, as shown in Table 4.

Patients with heart failure had 3.06 times the odds of death compared to those without (aOR: 3.06, 95% CI: [1.56, 6.32], *p*–value = 0.001). After stratification, heart failure was associated with 3.95 times the odds of death among those with HIV compared to those without HIV, and 2.64 times the odds of death among those without HIV. The interaction *p*-value, however, was not statistically significant (*p*-value = 0.465), indicating that the odds of mortality associated with heart failure showed no statistical evidence of effect modification by HIV infection.

Patients with acute myocardial infarction (AMI) had 5.83 times the odds of death compared to those without AMI. This association remained significant after adjusting for confounders (aOR: 9.07, 95% CI: [4.55, 18.77], *p* < 0.001). Stratification by HIV status showed a greater relative odds of death in patients living with HIV with AMI (OR: 10.23) compared to the non-HIV group (OR: 7.23). Although differences in all-cause mortality due to AMI across HIV status suggest possible effect modification, this interaction between AMI and HIV was not statistically significant (*p-*value = 0.613).

Patients with stroke had 2.5 times the odds of mortality compared to those without stroke (aOR: 2.5, 95% CI: [1.15, 5.42], *p*–value = 0.02). When stratified by HIV status, the observed relative odds of mortality among patients living with HIV (aOR: 1.84) and HIV-negative (aOR: 2.35) were not statistically significant. There was no statistical evidence of interaction between stroke and HIV infection in determining mortality difference (*p*-value = 0.615).

Overall, while adverse cardiac events (heart failure, AMI, and stroke) were associated with high odds of mortality in CVD patients with HIV, there was no statistical evidence of interaction between HIV infection and these events in determining mortality outcomes.

**Table 4:** Crude and adjusted odds ratios (and 95% CI), for the association between components of MACE and odds of Mortality, stratified by HIV status

Models	Crude Analyses (All CVD Patients) (*N* = 2127)		Adjusted Analyses (All CVD Patients) (*N* = 2127)		Stratification	
					CVD Patients Living with HIV **(** ***N*** ** = 292)**	CVD Patients without-HIV **(** ***N*** ** = 1835)**
	**OR** **95% CI**	***p*** **Value**	**OR** **95% CI**	***p*** **Value**	**OR** **95% CI**	**OR** **95% CI**
Heart Failure ^ℛ^						
Yes	3.70 [2.86, 4.83]	< 0.001	3.06 [1.56, 6.32]	0.001	3.95 [1.26, 14.50]	2.64 [1.08, 6.96]
Acute MI ^¥^						
Yes	5.83 [4.58, 7.44]	< 0.001	9.07 [4.55,18.77]	< 0.001	10.23 [3.12, 38.39]	7.23 [2.91,18.90]
Stroke ^€^						
Yes	2.57 [1.99, 3.31]	< 0.001	2.50 [1.15, 5.42]	0.020	1 0.84 [0.48, 7.07]	2.35 [0.86, 6.37]
ANY MACE^Ω^						
Yes	3.70 [2.86, 4.84]	< 0.001	3.04 [1.54, 6.27]	0.001	3.95 [1.26, 14.50]	2.58 [1.05, 6.82]
Interaction Terms						
Heart Failure * HIV		-		0.465		
Acute MI * HIV		-		0.613		
Stroke * HIV		-		0.615		

MACE: Major Adverse Cardiovascular Events, OR: odds ratio, CI: confidence interval, AMI: Acute Myocardial Infarction

ℛ Adjusted for: Sex, Age category, Cigarettes, Dyslipidemia, Antiplatelet medicines, Hypertension, Diabetes mellitus, Atrial fibrillation, Alcohol, and HIV status

ℬ Model with HIV variable interaction terms included

¥Adjusted for: Sex, Age category, Cigarettes, Dyslipidemia, Antiplatelet medicines, Hypertension, Diabetes Mellitus, Atrial fibrillation, Alcohol, and HIV status

€ Adjusted for: Sex, Age category, Cigarettes, Dyslipidemia, Antiplatelet medicines, Hypertension, Atrial fibrillation, Alcohol, and HIV status

Ω Adjusted for: Sex, Age category, Cigarettes, Dyslipidemia, Antiplatelet medicines, Hypertension, DM, Atrial fibrillation, Alcohol, HIV status, and Diabetes Mellitus

CI: Confidence Intervals

## Discussion

Our study shows all-cause mortality among hospitalized patients with cardiovascular disease patients living with and without HIV, in two northern Uganda hospitals. The cumulative incidence of all-cause mortality was higher among patients living with HIV (26.0%) than among those without HIV (15.8%). We also observed that patients living with HIV had 130.0% greater relative odds of mortality compared to HIV-seronegative individuals (aOR: 2.3, 95% CI: [1.32, 3.98]) after adjusting for confounders. This trend aligns with prior research in other regions, which consistently reports elevated CVD risk among individuals living with HIV [25, 33, 46, 47]. 

We observed a higher proportion of female (60.5%) than males (39.5%) patients hospitalized with CVD. These differences likely reflect hospitalization patterns, healthcare utilization, and health-seeking behavior, rather than differences in CVD, MACE, or mortality risks. In this region, females typically exhibit better health-seeking behavior than males. For instance, in Kampala, Uganda, men had 102.0% greater relative odds of hypertension than females, yet more females sought care [48]. Similarly, in rural Uganda, among people living with HIV, women receiving ART were three times more likely to be hospitalized than men, although men faced nearly four times the risk of mortality [49]. Despite the high burden of CVD and HIV comorbidity, research in low- and middle-income countries (LMICs), including sub-Saharan Africa, remains limited. LMICs account for 57.1% of global CVD deaths, yet contribute only 2.8% to global CVD research output [40]. This disparity underscores the need for increased investment in CVD research and a shift toward preventive strategies in these regions. Our findings highlight the critical public health challenge posed by this CVD-HIV comorbidity, emphasizing the need for integrated, resource-appropriate prevention and management interventions.

Among patients with major adverse cardiac events, those living with HIV had a higher mortality risk (38.1%) than seronegative patients (17.8%). Adjusted models showed increased odds of heart failure and stroke in patients living with HIV. Despite the high odd of mortality, there was no statistically significant interaction observed (non-significant *p*-values). This suggests that HIV infection contributes to long-term CVD risk and predisposes patients to adverse cardiac events (stroke and heart failure), leading to subsequent hospitalization with MACE. In addition, although patients living with HIV who experienced MACE had higher in-hospital mortality, there was no statistical evidence of effect modification by HIV status. No evidence for effect modification suggests that, the joint effects of HIV and MACE does not increase mortality beyond what would be expected from their individual effects [41], therefore, no evidence of biological synergistic or antagonistic interaction on either the additive or multiplicative scale during the acute phase of hospitalization [50]. In addition, our findings in patients with HIV and AMI suggest potential effect modification, as evidenced by large differences in the effect estimates (odds ratios) observed in stratified analyses. This suggests that the effect of AMI on all-cause mortality may differ by HIV status, although the interaction was not significant (*p*–value = 0.613). The lack of statistical significance for the interaction term does not provide conclusive evidence for the absence of interaction. This is because *p*–values are affected by effect estimate size, sample size, and variance in the data. Therefore, this could explain the non-significant *p*–value, despite the large differences in stratum-specific odds ratios compared to the odds ratio of the combined effect. Our findings were like many other studies that demonstrated an increased risk of major adverse events, and a differentially high risk of death in patients living with HIV. These include stroke [13–15], heart failure [17, 19], and acute myocardial infarction [51–55]. Other studies showed a lack of evidence for increased risk of death among patients with MACE and living with HIV [56]. The increased risk for CVD and MACE has been attributed to the impact of HIV on the cardiovascular system through a long-standing dysregulated inflammatory process [22, 26, 57], increased risk of atherosclerosis [33], endothelial dysfunction [58], and associated metabolic abnormalities [59]. However, the interaction between HIV and MACE affecting outcomes and prognosis requires further studies as this could optimize patient management.

The traditional Framingham risk factors for CVD, which contribute substantially to global morbidity and mortality, were evaluated in this study [60]. We examined the distribution of these factors and their association with the odds of all-cause mortality among patients with and without HIV infection. Nonmodifiable factors included sex, age, and family history of CVD, while modifiable lifestyle factors included smoking and alcohol use. Except for alcohol use (aOR: 0.95, 95% CI: [0.46 to 1.88], *p =* 0.898), these demographic factors were associated with higher mortality, with the HIV group experiencing greater relative odds than those without HIV. We also evaluated clinical CVD risk factors, including diabetes mellitus, hypertension, dyslipidemia, and chronic kidney disease, along with potential confounders such as antiplatelet use, atrial fibrillation, and dilated cardiomyopathy. Overall, these CVD risk factors were more prevalent among PLWH than those without HIV, consistent with earlier studies [9, 47, 61, 62]. Similarly, the relative odds of mortality were higher among CVD patients living with HIV.

Several mechanisms contribute to the increased risk of CVD in PLWH. These arise from the combined effects of traditional CVD risk factors [60], chronic inflammation, immune dysfunction, and treatment–related metabolic changes, even with effective ART [63], promote a persistent proatherogenic state driven by ongoing immune activation and direct endothelial injury [23]. Additional pathways include extracellular-vesicle-mediated vascular damage [24], coagulopathy and thrombosis during immune reconstitution or uncontrolled viremia, and marked dyslipidemia compared to HIV negative individuals [22, 26, 64–67]. Hypertension is a major driver of CVD in PLWH, and is strongly associated with AMI, stroke, and a substantial proportion of cardiac deaths [66]. Among PLWH, hypertension risks include HIV itself, genetic susceptibility, ART-related changes in body composition, immune activation, and ART [68]. Effective management of hypertension is therefore central to preventing CVD and MACE in this population. Overall, hypertension and other traditional CVD risk factors are more frequent among PLWH and are associated with higher mortality risk [65, 68, 69]. To guide prevention efforts, the 2025 European AIDS Clinical Society guidelines recommend a risk-based approach that includes statin therapy for primary prevention of atherosclerotic disease, blood pressure control, glucose monitoring, kidney disease management, and optimization of ART [70]. 

To prevent CVD and reduce related adverse events, screening and assessing PLWH provide a crucial opportunity to identify and manage risk factors early. This requires a multifaceted approach, including cardiovascular evaluation within HIV care and the use of CVD risk-prediction tools to guide timely interventions. However, cardiovascular risk factors, risk prediction models, and assessment in the context of HIV infection remain under-reported. A systematic review and meta-analysis in 2023, included 13 articles describing seventeen CVD risk models in HIV, none was in sub-Saharan Africa or the Asian region [71]. The necessity for a CVD risk assessment model tailored to this high-HIV-burden demographic is evident, as models from other settings may not perform optimally in different environments [71–73]. This is because many CVD risk prediction models were developed in non-HIV populations but have been adopted for use in HIV populations. Consequently, they underperformed when applied to HIV-positive individuals, showing moderate discrimination ability and underpredict CVD risk [71, 73]. This underperformance likely reflects regional and population differences in health determinants, leading to model optimism, where models perform better on training data than on new, unseen data [74]. Moreso, these models rely on traditional risk factors while overlooking HIV-specific inflammation and immune activation [75]. Furthermore, despite the increased risk for CVD and MACE, people living with HIV do not receive comparable CVD screening or care as their seronegative counterparts [76]. This is common in resource-limited settings where guidelines for screening CVD are not operationalized in routine comprehensive HIV care. Paradoxically, exposure to ART was reported as a risk factor for CVD [51, 77], as preventive in others [52, 78], while others showed no association [7, 78, 79]. Therefore, further studies on risk factors are needed to design optimal CVD screening tools tailored to HIV settings. This could help reduce the incidence of MACE and the associated morbidity and mortality.

Because HIV patients with major adverse events have a high risk of death upon hospitalization, preventing CVD in individuals with HIV remains a plausible public health intervention to curb this mortality. Other than the traditional CVD risk factors, low CD4 count, high HIV viral load, cumulative antiretroviral use as well as exposure to certain regimens have been reported as risk factors for MACE [51, 53, 55, 78]. This study did not evaluate risk factors for developing major adverse cardiovascular events among HIV patients. This was beyond the scope of our study, albeit the observed high risk of composite components of major adverse cardiac events at hospitalization.

Lastly, mortality among CVD patients hospitalized with major adverse cardiovascular events may follow a different course in the medium term (30–90 days) and long term (6 months to years) depending on HIV-status [80–82]. Our results are limited to in-hospital outcomes, and the association between patients living with and without after discharge may differ, underscoring the need for extended follow-up to fully characterize CVD outcome trajectories.

### Study strengths

The study has several strengths, importance, and contributions to the body of knowledge and policy. It utilized large data sources from two large hospitals in the region. We examined real-world CVD outcomes for patients living with HIV in a primary care setting, typical of resource-limited sub-Saharan Africa with high HIV rates. We demonstrated that the overall odds of all-cause mortality were higher among patients with HIV–CVD comorbidities compared to HIV-seronegative patients. This provides information for future studies or to design CVD risk prediction tools for the prevention of CVD in the sub-group with HIV. There was no statistically significant interaction between HIV infection and heart failure or stroke; however, there was evidence of an interaction for AMI. This has policy implications for targeting primary prevention of CVD among patients with HIV or preventing CVD-related adverse events from occurring than expect to improve outcomes during hospitalization. This may include ensuring appropriate adherence to lifestyle modification and early management of HIV infection to avert the occurrence of heart failure, AMI, and strokes in this population with high HIV prevalence. These findings are generalizable to hospitalized patients with cardiovascular disease in settings with a high prevalence of HIV infection, particularly in sub-Saharan Africa, and underscore the need for integrated HIV and cardiovascular care strategies in such regions.

### Study limitations

This study encountered limitations inherent to the retrospective nature of the study design, including incomplete or missing records and variables collected for purposes other than this study.

For example, clinical diagnoses and test results recorded during routine care depend on clinicians’ assessments based on symptoms, biomarkers, imaging, and prior records from the national referral hospital (Uganda Heart Institute). Consequently, detailed patient categorization was not feasible, even though disease severity can affect outcomes differently. For instance, advanced AMI classification (e.g., STEMI vs. NSTEMI or type 1–5 AMI) may require different interventions and lead to different outcomes [83]. However, these data were unavailable due to limited access to advanced cardiac services in this resource-limited setting, despite their importance in evaluating mortality among patients with adverse cardiovascular events. This limitation is not unique to our study setting but is a common finding in real-world healthcare settings in sub-Saharan Africa.

Secondly, the study was limited to in-hospital outcomes, which may not fully represent the source population. Mortality data excluded pre- and post-hospitalization outcomes due to a lack of access; therefore, the results are generalized only to hospitalized CVD patients in resource-limited settings in sub-Saharan Africa.

Thirdly, subgroup analyses for HIV-related factors like CD4 counts, ART use and duration, and HIV-viral load tests, which could affect mortality outcomes, were not possible due to missing records. These represent residual or unmeasured confounding factors specific to HIV that may have influenced the observed associations. For example, markers of HIV disease severity, such as CD4 and HIV-viral load in advanced HIV disease [84]. These are often not captured, particularly during this test-and-treat era, where ART is initiated without requiring CD4 or HIV viral load tests [85]. Accordingly, these limitations warrant interpretation of our findings in the context of the presented results.

Fourth, we excluded 48 patient files (2.2%) due to missing or incomplete records, which may introduce minimal selection bias. Selection bias due to differential referrals likely occurred, as the study included only patients treated at two regional hospitals, excluding those from lower-level centers who lacked funds or transportation to reach these facilities. These hospitalized patients may have been systematically different, likely sicker, than those managed elsewhere. Furthermore, the predominance of females (60.5%) among hospitalized CVD patients may not reflect the actual distribution of the source population, potentially introducing selection bias. This could have led to differential or non-differential selection bias, either underestimating or overestimating the effect estimates. However, it is unlikely that the observed effect estimates for hospitalized patients are significantly biased, as any potential bias would likely not explain the substantial magnitude of the observed effect sizes.

Additionally, only patients who survived to reach the hospital were included, introducing survivor bias. Most patients’ attendants in this region declined autopsies, meaning we could not access data on cardiac-specific deaths. The final diagnoses were based on clinical, laboratory, and other available investigations, which were not universally available to all patients with CVD. This could have led to non-differential misclassification of exposures, biasing effect estimates toward the null, and potentially underestimating the effects, leading to incorrect interpretations and misleading conclusions. However, any small observed effect estimates would likely be clinically significant, as the true estimates would likely be larger if exposure misclassification were absent.

Hospitalized patients, especially those with HIV and CVD, may have had competing risks of mortality based on therapy or interventions received, and other unmeasured covariates, such as malnutrition, medications received, monitoring, and pre- and post-hospitalization interventions, that were not assessed. These factors, including residual confounding factors, could have influenced the observed findings. In addition, there were small sample sizes in the HIV positive group, limiting explorative and robust subgroup analyses. These covariates could impact the interpretation of the study findings. For example, only 56/292 (19.2%) of CVD patients living with HIV were on antiplatelet therapy compared to 672/1,835 (36.6%) of their HIV negative counterparts. However, adherence to the therapy could impact the findings and yet it was unmeasured retrospectively. Therefore, despite evidence that antiplatelet use reduces adverse cardiovascular events, differences in adherence and interventions received could impact the outcome, and we acknowledge this limitation in our study because stratification by platelets level was impractical [86–88]. Random errors were likely minimal, as the confidence intervals for the effect estimates were narrow and precise except for AMI, which may be due to the small sample size.

Lastly, we considered logistic regression analysis because it’s suitable for modeling multiple variables and complex models, and it provides stables estimate when modeling interaction terms for effect modification. When the rare disease assumption is met (outcome < 10%), odds ratios (ORs) approximate risk ratios (RRs). The limitation is that when the outcome is common, as in our study, odds ratios overestimates the magnitude of association relative to risk ratios, although the effect estimates remain unbiased [89]. Secondly, risk ratios are not estimated directly for direct interpretation. Log-binomial regression, as an alternative approach, also faces convergence concerns when outcome prevalence becomes high or when continuous covariates or interaction terms are included in the model as in our study, which limits its utility [90]. Although our outcome variable was binary, modified Poisson regression, which assumes a log-linear relationship between the outcome risk and explanatory variables, is more suitable for heterogeneous outcomes because it converges reliably regardless of model complexity, outcome prevalence, or number of predictors. It also estimates risk ratios directly rather than odds ratios, providing valid estimates in a broader context than alternative methods [91]. Therefore, despite these limitations, logistic regression remains appropriate for modeling effect modification using interaction terms, although results must be interpreted with caution and in the context of outcome prevalence. Specifically, odds ratios should not be misconstrued as risk ratios, as they overestimate the magnitude of association when outcomes are common [92, 93]. Lastly, we expect that the estimated associations are likely unbiased and valid within the logistic regression framework; however, they reflect associations rather than causal relationships and should therefore be interpreted with caution.

Despite these limitations presented, the study provides valuable findings in this setting that could inform future research in sub-Saharan Africa.

## Conclusion

Our study found that cardiovascular disease patients living with HIV had a higher incidence proportion of all-cause mortality compared to those without HIV. Similarly, among patients hospitalized with major adverse cardiovascular events, mortality was higher in those co-infected with HIV compared to those without HIV. However, HIV status did not modify the odds of all-cause mortality during hospitalization, indicating no effect modification. In other words, HIV infection had no additional effect on the already high risk of all-cause mortality observed with MACE during hospitalization, a unique finding in this study. The high incidence of MACE and the disproportionate mortality burden among CVD patients with HIV warrants further studies to understand the pathophysiological mechanisms and associated factors, including both medium- and long-term post hospitalization. Therefore, the knowledge of the interaction between HIV exposure and CVD among patients in sub-Saharan Africa may improve outcomes and prevention strategies.

## Supplementary Information

Below is the link to the electronic supplementary material.

Supplementary Material 1

Supplementary Material 2

## Data Availability

Data were de-identified in compliance with HIPAA regulations and available in an open-access repository [94].

## Abbreviations

AMI: Acute Myocardial Infarction
aOR: Adjusted Odds Ratio
ART: Antiretroviral Therapy
CI: Confidence Interval
CKD: Chronic Kidney Disease
CS: Cigarette Smoking
CT: Computer Tomography
CVD: Cardiovascular Disease
CD4: Cluster of differentiation 4
DAG: Directed Acyclic Graph
DCMP: Dilated Cardiomyopathy
DM: Diabetes Mellitus
ECG: Electrocardiograph
HIV: Human Immunodeficiency Virus
HT: Hypertension
IQR: Interquartile Range
LMICs: Low- and middle-income countries
LRRH: Lira Regional Referral Hospital
LUH: Lira University Hospital
MACE: Major Adverse Cardiovascular Event
MRI: Magnetic Resonance Imaging
OR: Odds Ratio
PLWH: Persons Living with Human Immunodeficiency Virus
RHD: Rheumatic Heart Disease
WHO: World Health Organization
