# Supplementary material for: Major adverse cardiovascular events, morbidity, and mortality, among people living with and without HIV in two northern Uganda hospitals
Source: BMC Infect Dis. 2026 Feb 14;26:609. doi: 10.1186/s12879-026-12895-6 (PMC13011330; doi:10.1186/s12879-026-12895-6)
Supplement: Supplementary file 1 — Supplementary Material 1 [file 12879_2026_12895_MOESM1_ESM.zip › SUPPORTING_DOC_DAG_STROBE/DAG.PDF]

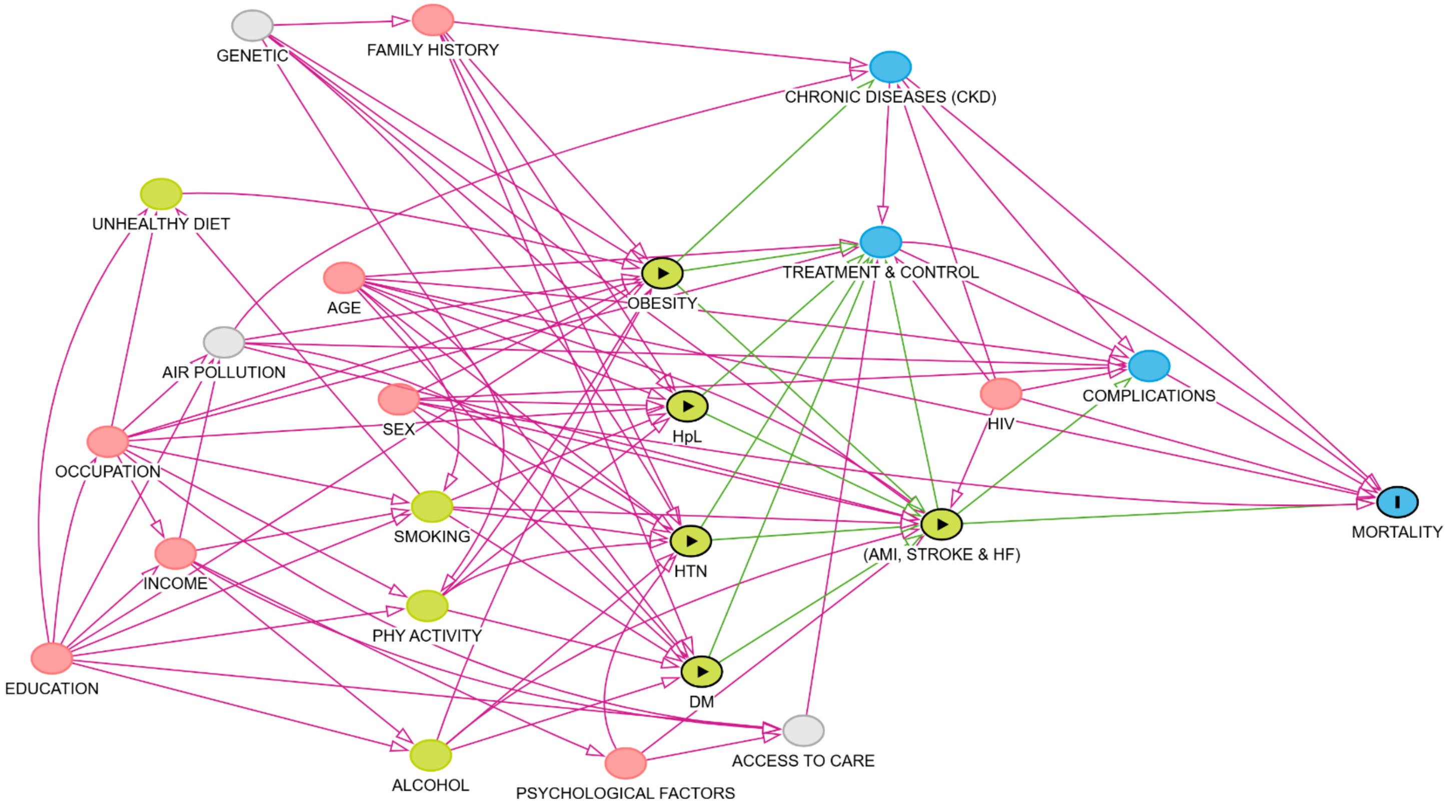

Figure S1: TRUE DAG, unadjusted (View Mode: Normal)

Figure S1 represents the unadjusted DAG, assuming it accurately reflects the actual causal structure of the real-world scenario based on current literature.
DM-Diabetes mellitus, PHY – Physical activity, HTN - Hypertension, AMI- Acute myocardial infarction, HF – Heart Failure, HpL – Hyperlipidemia, CKD – chronic kidney disease, and HIV – Human Immunodeficiency Virus

Legend Key:

exposure
outcome
ancestor of exposure
ancestor of outcome
ancestor of exposure *and* outcome
adjusted variable
unobserved (unmeasured)
causal path
biasing path

Dagitty.net: DAG Code (Exposure, AMI, Stroke and Heart Failures, and outcome, Mortality)

```
dag {
"(AMI, STROKE & HF)" [exposure,pos="0.307,0.478"]
"ACCESS TO CARE" [latent,pos="-0.009,1.257"]
"AIR POLLUTION" [latent,pos="-1.335,-0.209"]
"CHRONIC DISEASES (CKD)" [pos="0.190,-1.249"]
"FAMILY HISTORY" [pos="-0.857,-1.426"]
"PHY ACTIVITY" [pos="-0.870,0.786"]
"PSYCHOLOGICAL FACTORS" [pos="-0.416,1.380"]
"TREATMENT & CONTROL" [pos="0.168,-0.588"]
"UNHEALTHY DIET" [pos="-1.479,-0.769"]
AGE [pos="-1.060,-0.453"]
ALCOHOL [pos="-0.862,1.350"]
COMPLICATIONS [pos="0.782,-0.120"]
DM [exposure,pos="-0.242,1.034"]
EDUCATION [pos="-1.729,0.984"]
GENETIC [latent,pos="-1.270,-1.405"]
HIV [pos="0.443,-0.015"]
HTN [exposure,pos="-0.267,0.541"]
HpL [exposure,pos="-0.275,0.032"]
INCOME [pos="-1.446,0.588"]
MORTALITY [outcome,pos="1.350,0.394"]
OBESITY [exposure,pos="-0.332,-0.470"]
OCCUPATION [pos="-1.601,0.166"]
SEX [pos="-0.935,0.006"]
SMOKING [pos="-0.859,0.411"]
"(AMI, STROKE & HF)" -> "TREATMENT & CONTROL"
"(AMI, STROKE & HF)" -> COMPLICATIONS
"(AMI, STROKE & HF)" -> MORTALITY
"ACCESS TO CARE" -> "TREATMENT & CONTROL"
"AIR POLLUTION" -> "(AMI, STROKE & HF)"
"AIR POLLUTION" -> "CHRONIC DISEASES (CKD)" [pos="-1.104,-0.773"]
"AIR POLLUTION" -> COMPLICATIONS
"AIR POLLUTION" -> HTN [pos="-0.878,-0.166"]
"AIR POLLUTION" -> OBESITY
"CHRONIC DISEASES (CKD)" -> "TREATMENT & CONTROL"
"CHRONIC DISEASES (CKD)" -> COMPLICATIONS [pos="0.472,-0.655"]
"CHRONIC DISEASES (CKD)" -> MORTALITY [pos="0.657,-0.647"]
"FAMILY HISTORY" -> "CHRONIC DISEASES (CKD)"
"FAMILY HISTORY" -> DM
"FAMILY HISTORY" -> HTN
"FAMILY HISTORY" -> HpL
"FAMILY HISTORY" -> OBESITY
"PHY ACTIVITY" -> DM
"PHY ACTIVITY" -> HTN [pos="-0.704,0.575"]
"PHY ACTIVITY" -> HpL
"PHY ACTIVITY" -> OBESITY [pos="-0.734,0.592"]
"PSYCHOLOGICAL FACTORS" -> "(AMI, STROKE & HF)"
"PSYCHOLOGICAL FACTORS" -> "ACCESS TO CARE"
"PSYCHOLOGICAL FACTORS" -> HTN [pos="-0.533,1.047"]
"TREATMENT & CONTROL" -> COMPLICATIONS
"TREATMENT & CONTROL" -> MORTALITY [pos="0.584,-0.584"]
"UNHEALTHY DIET" -> OBESITY [pos="-1.093,-0.794"]
AGE -> "(AMI, STROKE & HF)" [pos="-0.101,-0.061"]
AGE -> "PHY ACTIVITY" [pos="-0.482,-0.023"]
AGE -> "TREATMENT & CONTROL"
AGE -> COMPLICATIONS [pos="-0.484,-0.394"]
AGE -> DM
AGE -> HTN
AGE -> HpL
AGE -> MORTALITY
AGE -> SMOKING [pos="-0.713,0.023"]
ALCOHOL -> "(AMI, STROKE & HF)" [pos="-0.471,0.798"]
ALCOHOL -> DM
ALCOHOL -> HTN
ALCOHOL -> OBESITY [pos="-0.707,0.617"]
COMPLICATIONS -> MORTALITY
DM -> "(AMI, STROKE & HF)"
DM -> "TREATMENT & CONTROL"
EDUCATION -> "ACCESS TO CARE"
EDUCATION -> "AIR POLLUTION"
EDUCATION -> "PHY ACTIVITY"
EDUCATION -> "UNHEALTHY DIET" [pos="-1.835,-0.031"]
EDUCATION -> ALCOHOL
EDUCATION -> INCOME
EDUCATION -> OBESITY [pos="-0.911,0.154"]
EDUCATION -> OCCUPATION [pos="-1.702,0.436"]
```

```
EDUCATION -> SMOKING
GENETIC -> "(AMI, STROKE & HF)"
GENETIC -> "FAMILY HISTORY"
GENETIC -> DM [pos="-0.821,-0.196"]
GENETIC -> HTN [pos="-0.694,-0.668"]
GENETIC -> HpL [pos="-0.620,-0.575"]
GENETIC -> OBESITY
HIV -> "(AMI, STROKE & HF)"
HIV -> "CHRONIC DISEASES (CKD)"
HIV -> "TREATMENT & CONTROL"
HIV -> COMPLICATIONS
HIV -> MORTALITY
HTN -> "(AMI, STROKE & HF)"
HTN -> "TREATMENT & CONTROL"
HpL -> "(AMI, STROKE & HF)"
HpL -> "TREATMENT & CONTROL"
INCOME -> "ACCESS TO CARE" [pos="-0.827,1.173"]
INCOME -> "AIR POLLUTION"
INCOME -> "PSYCHOLOGICAL FACTORS" [pos="-0.680,1.211"]
INCOME -> ALCOHOL
INCOME -> SMOKING
OBESITY -> "(AMI, STROKE & HF)"
OBESITY -> "CHRONIC DISEASES (CKD)"
OBESITY -> "TREATMENT & CONTROL"
OCCUPATION -> "ACCESS TO CARE" [pos="-0.854,1.165"]
OCCUPATION -> "AIR POLLUTION"
OCCUPATION -> "PHY ACTIVITY"
OCCUPATION -> "TREATMENT & CONTROL"
OCCUPATION -> "UNHEALTHY DIET"
OCCUPATION -> HpL
OCCUPATION -> INCOME
OCCUPATION -> OBESITY
OCCUPATION -> SMOKING
SEX -> "(AMI, STROKE & HF)"
SEX -> COMPLICATIONS
SEX -> DM
SEX -> HTN
SEX -> HpL
SEX -> MORTALITY [pos="0.630,0.461"]
SEX -> OBESITY
SMOKING -> "(AMI, STROKE & HF)"
SMOKING -> "UNHEALTHY DIET"
SMOKING -> DM
SMOKING -> HTN
SMOKING -> HpL
}
```

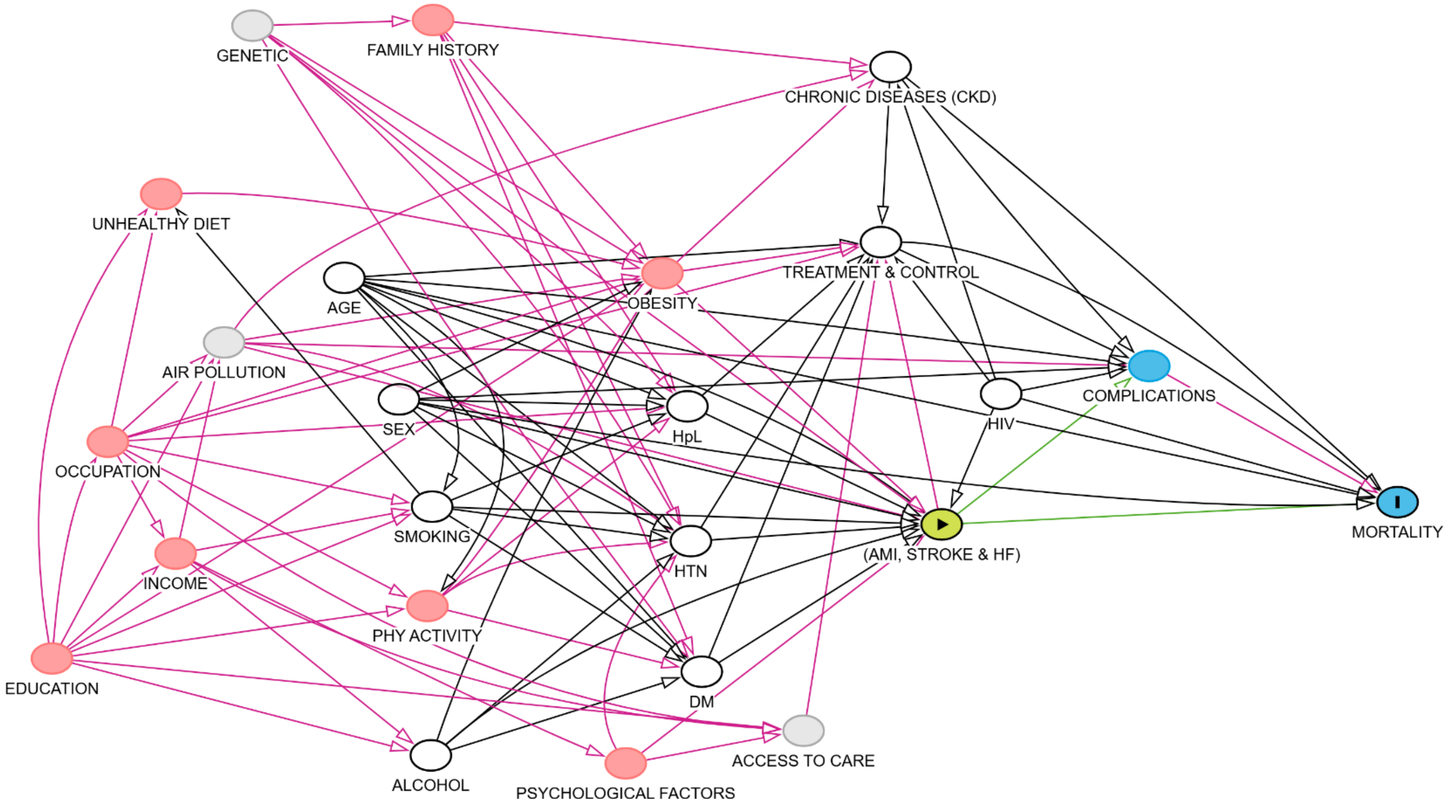

Figure S2: TRUE DAG, Adjusted Model
Shows the minimum sufficient set required for adjustment. However, the model may still be confounded by unmeasured confounders, which should also be considered for adjustment
DM-Diabetes mellitus, PHY – Physical activity, HTN- Hypertension, AMI- Acute myocardial infarction, HF – Heart Failure, HpL – Hyperlipidemia, CKD – chronic kidney disease, and HIV – Human Immunodeficiency Virus

Legend Key:
exposure
outcome
ancestor of outcome
ancestor of exposure *and* outcome
adjusted variable
unobserved (unmeasured)
causal path
biasing path

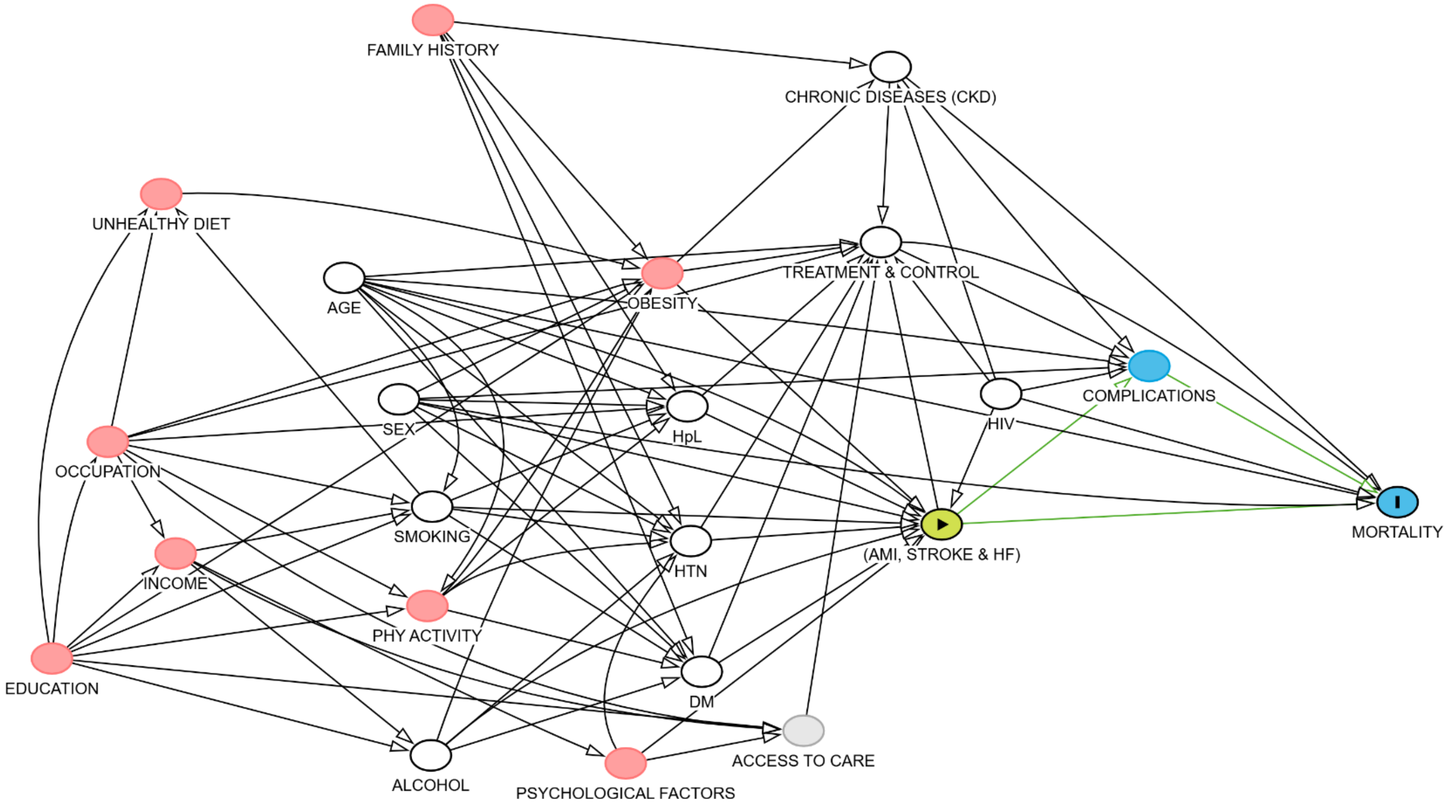

Figure S3: Final Hypothesized Model (Adjusted).
Figure S3 presents the adjusted hypothesized DAG, assumed to accurately represent the underlying causal structure and the minimum sufficient adjustment set required for adjustment.
DM-Diabetes mellitus, PHY – Physical activity, HTN- Hypertension, AMI- Acute myocardial infarction, HF – Heart Failure, HpL – Hyperlipidemia, CKD – chronic kidney disease, and HIV – Human Immunodeficiency Virus

Legend Key:
- exposure
- outcome
- ancestor of outcome
- ancestor of exposure *and* outcome
- adjusted variable (Selected)
- unobserved (unmeasured)
- causal path
