# Supplementary material for: Major adverse cardiovascular events, morbidity, and mortality, among people living with and without HIV in two northern Uganda hospitals
Source: BMC Infect Dis. 2026 Feb 14;26:609. doi: 10.1186/s12879-026-12895-6 (PMC13011330; doi:10.1186/s12879-026-12895-6)
Supplement: Supplementary file 1 — Supplementary Material 1 [file 12879_2026_12895_MOESM1_ESM.zip › SUPPORTING_DOC_DAG_STROBE/STROBE_CHECKLIST_CVD_HIV_v4-combined-PlosMedicine.docx]

STROBE Statement—checklist of items that should be included in reports of observational studies

|  | Item No. | Recommendation | Page  No. | Relevant text from manuscript | |
| --- | --- | --- | --- | --- | --- |
| **Title and abstract** | 1 | (*a*) Indicate the study’s design with a commonly used term in the title or the abstract | **Page 2** | | Retrospective Cohort Study |
|  |  | (*b*) Provide in the abstract an informative and balanced summary of what was done and what was found | **Page 2** | We aimed to determine the risk of all-cause mortality and morbidity in patients with CVD, comparing those with and without HIV infection, and whether mortality differed by HIV status during hospitalization for Major Adverse Cardiovascular Events (MACE) components (stroke, acute myocardial infarction (AMI), and heart failure) in northern Uganda.  Despite the increased incidence of MACE and mortality risk, in-hospital mortality did not differ by HIV status during hospitalization (no effect modification). | |
| Introduction | | | |  | |
| Background/rationale | 2 | Explain the scientific background and rationale for the investigation being reported | **Page 3-4** | Cardiovascular disease (CVD) morbidity and mortality is increasing globally, including among patients living with human immunodeficiency virus (HIV)  It is unclear if the presence of HIV infection in individuals hospitalized with major adverse cardiovascular events (stroke, MI and heart failure) could increase or modify the risk of in-hospital mortality. The uncertainty raises questions about whether prevention should focus on pre-hospitalization strategies, on improving care for hospitalized patients, or both if MACE-related mortality risk differs by HIV status. | |
| Objectives | 3 | State specific objectives, including any prespecified hypotheses | **Page 4-5** | We aimed to assess all-cause mortality and the risk of death from major adverse cardiovascular events (stroke, AMI, and heart failure) among patients with and without HIV hospitalized in two regional referral hospitals in northern Uganda. Additionally, the study assessed whether HIV infection modified the association between major adverse events and all-cause mortality during hospitalization. The study hypothesized that among patients hospitalized with major adverse cardiovascular events, comorbidity with HIV infection modifies and increases the risk of in-hospital mortality. Here we report on the outcomes of this retrospective cohort study which utilized data from two hospitals. We discuss the results and their implications for the prevention of CVD mortality in the context of HIV infection and major adverse cardiac events. | |
| Methods | | | |  | |
| Study design | 4 | Present key elements of study design early in the paper | **Page 5** | Retrospective Cohort Study | |
| Setting | 5 | Describe the setting, locations, and relevant dates, including periods of recruitment, exposure, follow-up, and data collection | **Page 5 - 6** | The study population was composed of patients hospitalized for any cardiovascular disease diagnoses at Lira Regional Referral Hospital (LRRH), and Lira University Hospital (LUH) between January 2015 to January 2022. We included all male and female adult patients aged 18 years and above, with confirmed HIV status, and any clinical diagnosis of CVD at the time of hospitalization.  In northern Uganda, LRRH and LUH tackle the region's high HIV and CVD burden. LRRH, a 350-bed government-owned hospital, located 340 km from Kampala, had 232,014 outpatient visits and 12,203 inpatient admissions in 2022  These two institutions have medical record departments, which we utilized as our data source | |
| Participants | 6 | (*a*) *Cohort study*—Give the eligibility criteria, and the sources and methods of selection of participants. Describe methods of follow-up  *Case-control study*—Give the eligibility criteria, and the sources and methods of case ascertainment and control selection. Give the rationale for the choice of cases and controls  *Cross-sectional study*—Give the eligibility criteria, and the sources and methods of selection of participants | **Page 5-6** | 2127 Patients Hospitalized with any cardiovascular disease | |
|  |  | (*b*) *Cohort study*—For matched studies, give matching criteria and number of exposed and unexposed  *Case-control study*—For matched studies, give matching criteria and the number of controls per case |  |  | |
| Variables | 7 | Clearly define all outcomes, exposures, predictors, potential confounders, and effect modifiers. Give diagnostic criteria, if applicable | **Page 6-7**  **Table 4 Page 18**  **(Confounders)** | Study population CVD patients  Exposure – 1) Heart Failure, 2) Acute MI, and 3) Stroke 4) Any MACE  Outcome Variable – Mortality  Effect modifier: Exposure to HIV infection (diagnosis)  Confounders: Based on DAG for specific or each exposure  e.g. for heart failure (Yes/No) and Outcome Mortality (Yes/No) model: the confounders based on the DAG were; Sex, Age category, Cigarettes, Dyslipidemia, Antiplatelet medicines, Hypertension, Diabetes mellitus, Atrial fibrillation, Alcohol, and HIV status | |
| Data sources/ measurement | 8* | For each variable of interest, give sources of data and details of methods of assessment (measurement). Describe comparability of assessment methods if there is more than one group | **Page 6-8** |  | |
| Bias | 9 | Describe any efforts to address potential sources of bias | **Page 7-8** | We adjusted for confounders in multivariable regressions. These confounders were selected *a priori* based on existing literature, and a directed acyclic graph (DAG) presented in supplementary Figures S1-S3. In addition, we used a 10% change in effect estimates (OR) to further identify confounders. We regressed mortality with each component of MACE and several covariates as linear predictors. Crude and adjusted OR were estimated for each component of MACE (exposure) models. Each model was fitted with their minimum sufficient sets of confounders. Confounders modeled included age, sex, smoking, diabetes, alcohol consumption, chronic kidney disease, dyslipidemia, hypertension, and history of cardiac disease. | |
| Study size | 10 | Explain how the study size was arrived at | **Page 5** | A total sample size of 2,127 patients was used, comprising all individuals hospitalized and treated during the study period who had a diagnosis of cardiac disease, with or without adverse events (heart failure, myocardial infarction, stroke, or any MACE). All eligible patients were included, except for 48 (2.2%) who were excluded due to missing or incomplete records (see Page 10). | |

Continued on next page

| Quantitative variables | 11 | Explain how quantitative variables were handled in the analyses. If applicable, describe which groupings were chosen and why | **Page 9-10** | We performed descriptive univariate statistical analyses using the Mann-Whitney U-test, a non-parametric statistical test utilized to compare the distributions of two independent groups. We described the study population using proportions, percentages, and median (interquartile range). The incidence of all-cause mortality among CVD patients was described using chi-squared analyses. We compared the risk of mortality among patients living with HIV and those without, describing mortality outcomes, stratified by HIV status.  Key Variables: were included in the study were categorized for easy interpretation and based on clinically meaningful categories. |
| --- | --- | --- | --- | --- |
| Statistical methods | 12 | (*a*) Describe all statistical methods, including those used to control for confounding | **Page 8-10** | We employed an unconditional logistic regression model to examine the association between all-cause mortality in CVD patients (outcome) and each component of major adverse cardiac events (exposure). Mortality, a binary variable coded as Death =1, and Alive=0 (reference group). The independent variables were Stroke (Yes=1, No=0), Heart failure (Yes=1, No=0), Acute myocardial infarction (Yes=1, No=0), and Any MACE (Yes=1, No=0). The absence of these conditions was considered as the reference group. After data cleaning, and checking for coding and data completeness, we examined data for outliers, missing values, and parametric assumptions (e.g. normality and independence of observations assumptions). We performed descriptive univariate statistical analyses using the Mann-Whitney U-test, a non-parametric statistical test utilized to compare the distributions of two independent groups. We described the study population using proportions, percentages, and median (interquartile range). The incidence of all-cause mortality among CVD patients was described using chi-squared analyses. We compared the risk of mortality among patients living with HIV and those without, describing mortality outcomes, stratified by HIV status.  The estimate of association was odds ratio (OR), and 95% confidence intervals (CI) for all-cause mortality, because the prevalence of death (outcome) was assumed to be less than 10%. We regressed mortality against each component of MACE (primary exposure) and several other covariates among CVD patients. Covariates were classified as binary or categorical (e.g. smoking categories). We adjusted for confounders in multivariable regressions. These confounders were selected *a priori* based on existing literature, and a directed acyclic graph (DAG) presented in supplementary Figures S1-S3. In addition, we used a 10% change in effect estimates (OR) to further identify confounders. We regressed mortality with each component of MACE and several covariates as linear predictors. Crude and adjusted OR were estimated for each component of MACE (exposure) models. Each model was fitted with their minimum sufficient sets of confounders. Confounders modeled included age, sex, smoking, diabetes, alcohol consumption, chronic kidney disease, dyslipidemia, hypertension, and history of cardiac disease. In all the analyses, statistical significance was defined as *a p*-value of <0.05 and a 95% confidence interval (5% two-sided alpha). |
|  |  | (*b*) Describe any methods used to examine subgroups and interactions | **Page 8-10** | We performed a stratified analysis, for each model for components of MACE (subgroups) to evaluate whether the effect of each composite component of MACE (stroke, AMI, and heart failure) on all-cause mortality differs by HIV status (HIV-positive/ negative) among CVD patients hospitalized with these adverse events. HIV interaction terms were included in the models (e.g. Stroke*HIV) to evaluate effect modification. |
|  |  | (*c*) Explain how missing data were addressed | **Page 9-10** | Patients with missing data (48/2,175; 2.2%) were excluded during data collection and analyses, this was because data were missing at random. |
|  |  | (*d*) *Cohort study*—If applicable, explain how loss to follow-up was addressed  *Case-control study*—If applicable, explain how matching of cases and controls was addressed  *Cross-sectional study*—If applicable, describe analytical methods taking account of sampling strategy | **Not Applicable** |  |
|  |  | (*e*) Describe any sensitivity analyses |  |  |
| Results | | | | |
| Participants | 13* | (a) Report numbers of individuals at each stage of study—e.g. numbers potentially eligible, examined for eligibility, confirmed eligible, included in the study, completing follow-up, and analysed | **Page 5** | 2,127 patients (All Patients Considered) |
|  |  | (b) Give reasons for non-participation at each stage |  | Not Applicable |
|  |  | (c) Consider use of a flow diagram |  | All patients considered |
| Descriptive data | 14* | (a) Give characteristics of study participants (eg demographic, clinical, social) and information on exposures and potential confounders | **Page 10-11** | Yes, refer to **Table 1. (Page 27)** |
|  |  | (b) Indicate number of participants with missing data for each variable of interest | **Page 9-10** | 48 patients (2.2%) of all patients recruited |
|  |  | (c) *Cohort study*—Summarise follow-up time (eg, average and total amount) |  | Retrospective cohort study |
| Outcome data | 15* | *Cohort study*—Report numbers of outcome events or summary measures over time | *Page 28*  *Page 29*  *Page 30* | *Table 2*  *Table 3*  *Table 4* |
|  |  | *Case-control study—*Report numbers in each exposure category, or summary measures of exposure |  |  |
|  |  | *Cross-sectional study—*Report numbers of outcome events or summary measures |  |  |
| Main results | 16 | (*a*) Give unadjusted estimates and, if applicable, confounder-adjusted estimates and their precision (eg, 95% confidence interval). Make clear which confounders were adjusted for and why they were included | *Page 29*  *Page 30* | ***Table 3***  ***Table 4*** |
|  |  | (*b*) Report category boundaries when continuous variables were categorized | Yes | *Table 3(Page 29)* |
|  |  | (*c*) If relevant, consider translating estimates of relative risk into absolute risk for a meaningful time period |  |  |

Continued on next page

| Other analyses | 17 | Report other analyses done—eg analyses of subgroups and interactions, and sensitivity analyses | **Page 10-14** | We conducted an adjusted multivariable analysis to assess the risk of mortality associated with major adverse cardiac events, stratified by HIV status, as shown in **Table 3, and Tablev4.** |
| --- | --- | --- | --- | --- |
| Discussion | | | | |
| Key results | 18 | Summarise key results with reference to study objectives | **Page 15-17** | Yes, summarized results and implications, with comparisons to other studies given |
| Limitations | 19 | Discuss limitations of the study, taking into account sources of potential bias or imprecision. Discuss both direction and magnitude of any potential bias | **Page 18-19** | Strengths and limitations of the study |
| Interpretation | 20 | Give a cautious overall interpretation of results considering objectives, limitations, multiplicity of analyses, results from similar studies, and other relevant evidence | **Page 15-17** | Yes, it provided for, point -by-point with references and other relevant evidence especially justifying the need for early preventive approach CVD and MACE than treatment. |
| Generalisability | 21 | Discuss the generalisability (external validity) of the study results | **Page 18 (Line 417-420)** | These findings are generalizable to hospitalized patients with cardiovascular disease in settings with a high prevalence of HIV infection, particularly in sub-Saharan Africa, and underscore the need for integrated HIV and cardiovascular care strategies in such regions. |
| Other information | |  | | |
| Funding | 22 | Give the source of funding and the role of the funders for the present study and, if applicable, for the original study on which the present article is based | **Page 20** | No Funding |

*Give information separately for cases and controls in case-control studies and, if applicable, for exposed and unexposed groups in cohort and cross-sectional studies.

**Note:** An Explanation and Elaboration article discusses each checklist item and gives methodological background and published examples of transparent reporting. The STROBE checklist is best used in conjunction with this article (freely available on the Web sites of PLoS Medicine at http://www.plosmedicine.org/, Annals of Internal Medicine at http://www.annals.org/, and Epidemiology at http://www.epidem.com/). Information on the STROBE Initiative is available at www.strobe-statement.org.
