# Supplementary material for: Major adverse cardiovascular events, morbidity, and mortality, among people living with and without HIV in two northern Uganda hospitals
Source: BMC Infect Dis. 2026 Feb 14;26:609. doi: 10.1186/s12879-026-12895-6 (PMC13011330; doi:10.1186/s12879-026-12895-6)
Supplement: Supplementary file 2 — Supplementary Material 2 [file 12879_2026_12895_MOESM2_ESM.pdf]

Appendix 1: DATA EXTRACTION TOOL

Major Adverse Cardiovascular Events, Morbidity, and Mortality, Among People Living with and without HIV in Two Northern Uganda Hospitals

A. Identification

1. Study ID: ……………………….
2. Inpatient ID: …………………….
3. Date of first Admission: ……..…/………/…………
4. Date of Outcome (Death, Discharge, or lost to follow up)….…./….……/…………
5. Age: ……………. Years
6. Sex:
   a. Male
   b. Female

B. Socio-demographic

7. Tribe:

(a) Lango	(b) Acholi	(c) Itesot	(d) Muganda
(e) Madi	(f) Alur	(g) Kumam	(h) Others

8. Marital Status

a) Single   b) Married   c) Separated   d) Divorced   e) Widowed   f) missing data

9. Religion

a) Catholic	b) Anglicans	c) Pentecostal	d) Moslem	e) Orthodox
f) Seventh Day	g) Jehovah witness		h) Atheist	i) missing data

10. District Physical Address

a) Lira	b) Oyam	c) Apach	d) Pader
e) Amolatar	f) Otuke	g) Kwania	h) Abim
i) Dokolo	j) Alebtong	k) Kole	Others……………..

11. Education Status

a) Primary	c)Institution	e) Higher Degree	g) Home trained
b) Secondary	d) University	f) No Formal Training	
g) Missing data			

12. Occupation

a) Unemployed b] self-employed c] formally employed d) student f) missing data

13. HIV status if Known
    a. Known Positive
    b. Known Negative
    c. Unknown
14. ART Use status if known HIV positive
    a. Yes
    b. No
    c. Missing data
15. Duration of ART use for HIV, date Started ……………………….
16. ART Adherence Category if documented

a) ART Naïve	b) ART Adherent	c) ART Defaulter	d) ART restarted after default	e) ART Exposed but not on ART	Missing data

17. HIV Immunological status
    a. CD4 Count……………………….…….cells/cc
    b. Viral load: …………………………. Copies/mls
    c. Missing data
18. Hepatitis B status on Admission
    a. Positive
    b. Negative
    c. Missing data
19. History of Herbs in the previous years
    a. Yes
    b. No
    c. Missing data
20. Alcohol consumption presently

a) i) Yes ii) No iii) missing data	
b) If yes, how long	…………………………… years

21. Diet type

a) Ground nut consumption	i) Yes	ii) No
b) Mushroom consumption	i) Yes	ii) No
c) missing data		

22. Type of Visit for Admission

a) Through OPD	b) Walk-in from home	c) Emergency Unit	d) Referral in

C. Clinical presentation (on first Admission)

History

23. **Cardiovascular System**

a) Lower Limb edema/swelling	a) Yes	b) No	c) Duration……(days)
b) Easy fatigability	a) Yes	b) No	c) Duration……(days)
c) Chest Pain	a) Yes	b) No	c) Duration……(days)
d) Shortness of breath	a) Yes	b) No	c) Duration……(days)
e) Palpitations	a) Yes	b) No	c) Duration……(days)
f) Collapse or syncope	a) Yes	b) No	c) Duration……(days)
g) Past Cardiovascular Disease	If yes, Type……………………………………..		

24. **Respiratory System**

a) Cough	a) Yes	b) No	c) Duration……(days)
b) Hemoptysis	a) Yes	b) No	c) Duration……(days)
c) Wheezing	a) Yes	b) No	c) Duration……(days)
d) Labored breathing	a) Yes	b) No	c) Duration……(days)
e) Granting	a) Yes	b) No	c) Duration……(days)
f) Past medical history of chest problems	If yes, type…………………………………………………..		

25. **Gastrointestinal and Liver**

a) Abdominal Pain	a) Yes	b) No	c) Duration……(days)
b) Abdominal distention	a) Yes	b) No	c) Duration……(days)
c) Vomiting	a) Yes	b) No	c) Duration……(days)
d) Yellow eyes	a) Yes	b) No	c) Duration……(days)
e) Vomiting Blood	a) Yes	b) No	c) Duration……(days)
f) Bloody stool	a) Yes	b) No	c) Duration……(days)
g) Diarrhea	a) Yes	b) No	c) Duration……(days)
h) Dysphagia	a) Yes	b) No	c) Duration……(days)
i) Odynophagia	a) Yes	b) No	c) Duration……(days)
j) Wasting	a) Yes	b) No	c) Duration……(days)
k) Itchy skin	a) Yes	b) No	c) Duration……(days)
l) Ingestion of herbs	a) Yes	b) No	c) Duration……(days)
m) Others symptoms	If yes type………..……………………		
n) Past Liver Disease	If yes Type……………………………………..		

26. **Social History**

a) Alcohol intake	a) Yes	b) No	c)Past use	d) ………(Months)
b) Smoking cigarettes	a) Yes	b) No	c)Past use	d) ………(Months)
c) Use of Drugs of recreation	a) Yes	b) No	c) Past use	d) ………(Months)
d) Multiple sexual partners	a) Yes	b) No	c) No partner	d) missing data

27. **Family Medical history**

a) Liver disease	a) Yes	b) No	c) missing data
b) Cardiac disease	a) Yes	b) No	c) missing data
c) Hypertension	a) Yes	b) No	c) missing data
d) Asthma or Allergies	a) Yes	b) No	c) missing data
e) Mental illness	a) Yes	b) No	c) missing data
f) Diabetes	a) Yes	b) No	c) missing data
g) Kidney disease	a) Yes	b) No	c) missing data
h) Cancers	a) Yes	b) No	c) missing data
i) Lung disease	a) Yes	b) No	c) missing data

28. **Musculoskeletal System**

a) Fever	a) Yes	b) No	c) missing data
b) Joint Pains	a) Yes	b) No	c) missing data
c) Joint swelling	a) Yes	b) No	c) missing data
d) Muscle Pains	a) Yes	b) No	c) missing data
e) Darkening of skin	a) Yes	b) No	c) missing data
f) Lightening of skin	a) Yes	b) No	c) missing data
g) Skin Rashes	a) Yes	b) No	c) missing data
h) Past medical history of skin/joint disease	If yes,<br>Type………………………………………………..		

29. **Central Nervous System**

a) Convulsions	a) Yes	b) No	c) missing data
b) Loss of consciousness/coma	a) Yes	b) No	c) missing data
c) Tremors	a) Yes	b) No	c) missing data
d) Over sleepiness	a) Yes	b) No	c) missing data
e) Confusion	a) Yes	b) No	c) missing data
f) Muscle wasting	a) Yes	b) No	c) missing data
g) Double vision	a) Yes	b) No	c) missing data
h) Hemiparesis/hemiplegia	a) Yes	b) No	c) missing data
i) Insomnia	a) Yes	b) No	c) missing data
j) History of Past CNS disease	If Yes<br>Type……………………………………….		

30. **Genitourinary System**

a) Dysuria	a) Yes	b) No	c) missing data
b) Genital Ulcer	a) Yes	b) No	c) missing data
c) Genital Discharge	a) Yes	b) No	c) missing data
d) Decreased Urine output	a) Yes	b) No	c) missing data
e) Polyuria	a) Yes	b) No	c) missing data
f) Past History of GUS	If Yes<br>Type……………………………………….		

31. **Past Medical History/general**

a) Herb Use	a) Yes	b) No	c) missing data
b) Trauma	a) Yes	b) No	c) missing data
c) Hepatitis B/C treatment	a) Yes	b) No	c) missing data
d) Ultrasound/Cardiac Echocardiogragphy done before<br>1) Yes 2) No. 3) Missing data	If yes<br>Type diagnosis…………………………		
e) Chronic drug Use before<br>1) Yes 2) No 3) Missing data	If Yes, Type Drug name…………………………………………		
f) Biopsy Done<br>1) Yes. 2) No 3) Missing data	If Yes<br>Type diagnosis………………………………		

D. Examination findings

32. **General Exam.**

a) Cyanosis
d) Jaundice
g) Dehydrated
k) Hyperpigmentation
b) Wasted
e) Pallor
h) Lymphadenopathy
l) Edema
c) Finger Clubbing
f) Febrile
i) Leukonychia
m) Koilonychia

33. **Vital Examination**

a) SPO2
d) Systolic BP
g) Urine output
j) RBS
b) Respiratory rate
e) Diastolic BP
h) Temperature
c) Pulse Rate
f) RBS
i) Glasgow Coma Scale

Specific System Examination

34. **Abdominal exam**

a) Ascites	a) Yes	b) No	c) missing data
b) Tenderness	a) Yes	b) No	c) missing data
c) Collaterals	a) Yes	b) No	c) missing data
d) Splenomegaly	a) Yes	b) No	c) missing data
e) Hepatomegaly	a) Yes	b) No	c) missing data
f) Abdominal Distention	a) Yes	b) No	c) missing data

35. **Cardiovascular System**

a) Blood pressure	Systolic…………	Mm/Hg
	Diastolic …………………	Mm/Hg
b) Pulse rate	………………………… Beats/min	
c) Murmurs	a) Yes	b) No
d) Edema	a) Yes	b) No
e) Fibrillation	a) Yes	b) No

36. **Respiratory System**

a) Respiratory Rate	…………………………Breaths/min		
b) Wheezes	a) Yes	b) No	c) missing data
c) Ronchi	a) Yes	b) No	c) missing data
d) Labored respiration	a) Yes	b) No	c) missing data
e) Effusion evidence	a) Yes	b) No	c) missing data
f) Emphysema evidence	a) Yes	b) No	c) missing data

37. **Musculoskeletal System.**

a) Joint Swelling	a) Yes	b) No	c) missing data
b) Hyperpigmented skin	a) Yes	b) No	c) missing data
c) Muscle atrophy	a) Yes	b) No	c) missing data

38. **Central Nervous System**

a) Neck rigidity	a) Yes	b) No	c) missing data
b) Planter reflex - positive	a) Yes	b) No	c) missing data
c) Babinskys sign positive	a) Yes	b) No	c) missing data
d) Hemiplegia - positive	a) Yes	b) No	c) missing data
e) Flapping tremors	a) Yes	b) No	c) missing data
f) Pinpoint pupils	a) Yes	b) No	c) missing data
g) Anisocorrhea	a) Yes	b) No	c) missing data
h) Coma	a) Yes	b) No	c) missing data
i) Glasgow coma scale	……………………… out of 15		

E. Laboratory Findings

39. **Complete blood count (CBC)**
    a. White cell counts…………………… x 10^3 cells/ul
    b. Lymphocytes………………… x 10^3 cells/ul
    c. Granulocytes………………... x 10^3 cells/ul
    d. Mid …………………………… x 10^3 cells/ul
    e. Hemoglobin……………………… g/dl
    f. MCV …………………………………… fl
    g. MCH …………………………………… pg
    h. Platelet Count …………………………x 10^3 cells/ul
40. **Current CD4 + count ………………………… cells/ul**
41. **Hepatitis B viral load ………………………… copies/ml**

42. **LIVER FUNCTION TEST (first visit)**

a) Total serum Protein	**………**	**g/l**	Missing data
b) Albumin, Alb	**……….**	**g/l**	Missing data
c) Bilirubin Total, Bil-T		**Umol/l**	Missing data
d) Bilirubin Direct, Bil-d	**…………**	**Umol/l**	Missing data
e) Alanine aminotransferase, ALT	**…………**	**u/l**	Missing data
f) Aspartate aminotransferase, AST	**…………**	**u/l**	Missing data
g) Alkaline phosphatase, ALP	**…………**	**u/l**	Missing data
h) Gama Glutamyltransferase, GGT	**…………**	**u/l**	Missing data
i) Prothrombin Time, PT	**…………**	**Seconds**	Missing data
j) International normalized ratio, INR	………..		

43. **BIOCHEMICAL TEST (last admission prior outcome)**

k) Total serum Protein	**………**	**g/l**	Date …………..
l) Albumin, Alb	**……….**	**g/l**	Date …………..
m) Bilirubin Total, Bil-T		**Umol/l**	Date …………..
n) Bilirubin Direct, Bil-d	**…………**	**Umol/l**	Date …………..
o) Alanine aminotransferase, ALT	**…………**	**u/l**	Date …………..
p) Aspartate aminotransferase, AST	**…………**	**u/l**	Date …………..
q) Alkaline phosphatase, ALP	**…………**	**u/l**	Date …………..
r) Gama Glutamyl transferase, GGT	**…………**	**u/l**	Date …………..
s) Prothrombin Time, PT	**…………**	**Seconds**	Date …………..
t) International normalized ratio, INR	………..		
u) Troponin – T/I	(specify)…………..		
v) HDL, High Density Lipoproteins		mg/dl	Date …………..
w) LDL, low Density Lipoproteins		mg/dl	Date …………..
x) Cholesterol, (random)		mg/dl	Date …………..
y) TRIG, triglycerides		mg/dl	Date …………..
z) HBA1C		%	Date …………..

44. **RENAL FUNCTION TEST**
   a. Serum Urea………………. mmol/1
   b. Serum Creatinine…………….umol/1
   c. Sodium……………………. mmol/l
   d. Potassium ………………. mmol/l

45. **Results of other investigation (CSF)**
   a. Done: 1) Yes 2) No
   b. If Yes
      i. Lymphocyte predominant 1] Yes … 2] No …
      ii. Neutrophil Predominant 1] Yes … 2] No …
      iii. India ink positive 1] Yes … 2] No …
      iv. CRAG LFA positive 1] Yes … 2] No …
      v. Neuro-toxoplasma IGG positive 1] Yes … 2] No …

vi. CSF not done 1] Yes … 2] No …
vii. CSF sugar ……………. Mg/dl

46. Blood sugar …………………….mg/dl
47. Chest X-Ray Done
    a. 1) Yes 2) No
    b.

a) Normal	b) Effusion	c) Cannon Balls	d) Cavities
e) Heterogeneous opacity	f) Pneumonia	g) Consolidation	h) Pneumothorax
i) Ground glass appearance	j) Cotton wool appearance	k) Milliary pattern	l) Non-specific

48. Urine Analysis Done:
    a. 1) Yes 2) No
    b. If Yes
        i. Protein
            1. Nil
            2. +1
            3. +2
            4. +3
    c. Urine SG
        i. Normal
        ii. Low
        iii. High
49. Serum LDH
    a. 1) Yes. Or 2) No
    b.
        i. Normal
        ii. low
        iii. High
50. Sputum for AFB (ZN)
    a. Done 1) Yes or 2) No
    b. If Yes
        i. Negative
        ii. positive
        iii. Sputum Gene Xpert
            1. a] Negative b] Positive
51. Serum RPR
    a. Done 1) Yes or 2) No
    b. If Yes 1) Negative 2) Positive
52. Serology

	Data available	Results if yes
**a)** Hepatitis A	a) Yes b) No	a) positive b) Negative
b) Hepatitis B	a) Yes b) No	a) positive b) Negative
c) Hepatitis C	a) Yes b) No	a) positive b) Negative
d) Hepatitis D	a) Yes b) No	a) positive b) Negative

e) Hepatitis E	a) Yes b) No	a) positive b) Negative
f) HIV	a) Yes b) No	a) positive b) Negative
g) Malaria RDT	a) Yes b) No	a) positive b) Negative

53. Abnormal Aspirates (Ascites) done for SBP
    a. Yes
    b. No
    c. Not done
54. Abdominal Ultrasonography (Liver)

a) Yes<br>b) No<br>c) missing data	If yes, specify liver findings	………………………………………………<br>………………………………………………

55. **Cardiac Echocardiography**

a) Yes<br>b) No<br>c) missing data	If yes, specify findings	………………………………………………<br>………………………………………………

56. **Electrocardiogram Test**

a) Yes<br>b) No<br>c) missing data	If yes, specify findings	………………………………………………<br>………………………………………………

57. **Treatment or Management done.**

a) Drugs	If Yes Specify………………………………………………………………	
b) Blood Transfusion	a) Yes	b) No
c) Morphine for palliation	a) Yes	b) No
d) Paracentesis	a) Yes	b) No
e) ART for HIV	If Yes, Specify	……………………………….
f) ART for Hepatitis B	If Yes, Specify	………………………………

58. **Diagnosis of Liver Disease**

a) Liver Cirrhosis	a) Yes	b) No
b) Alcoholic Liver Disease	a) Yes	b) No
c) Non-Alcoholic Liver Disease	a) Yes	b) No
d) Acute HBV infection	a) Yes	b) No
e) Hepatocellular Carcinoma	a) Yes	b) No
f) Drug-Induced Liver Injury-Herbs	a) Yes	b) No

g) Drug-Induced Liver Injury – Therapeutic drugs	a) Yes	b) No
h) Hepatitis A	a) Yes	b) No
i) Chronic Hepatitis B	a) Yes	b) No
j) Chronic Hepatitis C	a) Yes	b) No
k) Hepatitis D	a) Yes	b) No
l) Hepatitis E	a) Yes	b) No
m) Primary Biliary Cirrhosis	a) Yes	b) No
n) Primary Sclerosing Cholangitis	a) Yes	b) No
o) Autoimmune Liver Disease	a) Yes	b) No
p) Granulomatous Liver Disease	a) Yes	b) No
q) Secondary Liver Malignancy	a) Yes	b) No
r) Wilsons Disease	a) Yes	b) No
s) Alfa1-antitrypsin deficiency	a) Yes	b) No
t) Hemochromatosis	a) Yes	b) No
u) Idiopathic/Unknown etiology	a) Yes	b) No
v) Others Specify	………………………………………………………………..	

59. **Concurrent Diagnosis**

a) Other diagnosis present	a) Yes	b) No
b) If Yes, Specify	………………………………………………………………………	

60. **Other Medical disease(s) present/Diagnosis**

I. Cryptococcal meningitis	II. Cerebral Malaria	III. Urinary tract infection
IV. Tuberculosis meningitis	V. Neuro-toxoplasmosis	VI. Tuberculosis
VII. Bacterial Meningitis	VIII. Candida	IX. Hepatic encephalopathy
X. Viral meningoencephalitis	XI. Septicemia	XII. Pneumonia
XIII. HIV psychosis	XIV. Karposis sarcoma	XV. Malaria
XVI. Chronic Kidney disease	XVII. Asthma	XVIII. Pericarditis

XIX. Acute Kidney disease	XX. Rheumatic heart disease	XXI. Drug poisoning
XXII. Nephrotic syndrome	XXIII. Congestive heart failure	XXIV. Leuckemia
XXV. Cytomegalovirus	XXVI. Anemia	XVII. Lymphoma
XVIII. Sickle cell disease	XXIX. Bleeding PUD	XXX. Burkits lymphoma
XXXI. COPD	XXII. Hyperthyroidism	XXIII. Nephrolithiasis
XXIV. Delirium	XXV. Acute pancreatitis	XXVI. Cholelithiasis
XVII. Diabetes ketocidosis	XVIII. Chronic pancreatitis	XXIX. HMS
XL. Diabetes Hypoglycaemia	XLI. Cholecystitis	XLII. DVT
2) OTHERS	SPECIFY ………………………………………………………………………………	

61. **Complications during hospitalization**

	Response	If yes specify date of diagnosis
a) Heart Failure	a) Yes b) No	
b) Liver Failure	a) Yes b) No	
c) Renal Failure	a) Yes b) No	
d) Bleeding	a) Yes b) No	
e) Anemia	a) Yes b) No	
f) Encephalopathy	a) Yes b) No	
g) Varicose bleeding	a) Yes b) No	
h) Others	Specify …………………	

62. **Diagnosis of NCD**
    a. **Present**
    b. **Absent**
63. **If NCD present, Specify ………………………………………**

64. **Cardiac Outcomes**

	**Response**	**Date**
**1) Heart Failure**	a) Yes b) No	
**2) Acute Myocardial Infarction**	a) Yes b) No	
**3) Stroke**	a) Yes b) No	
**4) Dilated Cardiomyopathy**	a) Yes b) No	
**5) Severe Hypertension**	a) Yes b) No	

65. **Disposition**

	Response	Date
**6) Dead at Discharge**	a) Yes b) No	
**7) Alive at Discharged home**	a) Yes b) No	
**8) Referred for specialized care**	a) Yes b) No	
**9) Lost to Follow Up**	a) Yes b) No	
